# Assemblies of Polyacrylonitrile-Derived Photoactive Polymers as Blue and Green Light Photo-Cocatalysts for Cu-Catalyzed ATRP in Water and Organic Solvents

**DOI:** 10.3389/fchem.2021.734076

**Published:** 2021-08-13

**Authors:** Mingkang Sun, Francesca Lorandi, Rui Yuan, Sajjad Dadashi-Silab, Tomasz Kowalewski, Krzysztof Matyjaszewski

**Affiliations:** Department of Chemistry, Carnegie Mellon University, Pittsburgh, PA, United States

**Keywords:** ATRP, photocatalyst, polyacrylonitrile, carbon dot, luminescence, self-assembly

## Abstract

Photoluminescent nanosized quasi-spherical polymeric assemblies prepared by the hydrothermal reaction of polyacrylonitrile (PAN), *ht*-PLP_PAN_, were demonstrated to have the ability to photo-induce atom transfer radical polymerization (ATRP) catalyzed by low, parts per million concentrations of Cu^II^ complex with tris(2-pyridylmethyl)amine (TPMA). Such photo induced ATRP reactions of acrylate and methacrylate monomers were performed in water or organic solvents, using *ht*-PLP_PAN_ as the photo-cocatalyst under blue or green light irradiation. Mechanistic studies indicate that *ht*-PLP_PAN_ helps to sustain the polymerization by facilitating the activation of alkyl bromide species by two modes: 1) green or blue light-driven photoreduction of the Cu^II^ catalyst to the activating Cu^I^ form, and 2) direct activation of dormant alkyl bromide species which occurs only under blue light. The photoreduction of the Cu^II^ complex by *ht*-PLP_PAN_ was confirmed by linear sweep voltammetry performed under illumination. Analysis of the polymerization kinetics in aqueous media indicated even though Cu^I^ complexes comprised only 1–1.4% of all Cu species at equilibrium, they exhibited high activation rate constant and activated the alkyl bromide initiators five to six orders of magnitude faster than *ht*-PLP_PAN_.

## Introduction

Photocatalytic reactions play a profound role in various areas of chemical research (Zhu and Wang, 2017; Melchionna and Fornasiero, 2020). As alternatives to traditionally used transition metal complexes, organic photocatalysts (OPC) have received great attention, due to their low cost, highly tunable structures and photophysical properties (Romero and Nicewicz, 2016). More recently, organic photoactive nanostructured objects (OPNO) have emerged as promising materials owing to the growing interest in combining organic chemistry and nanotechnology (Han et al., 2018; Zhang et al., 2020). OPNO can span the range from covalently bonded “carbon dots” (Sun et al., 2006) to “polymer dots” (Zhu et al., 2015a; Zhu et al., 2015b; Tao et al., 2019; Xia et al., 2019) held together *via* non-covalent interactions. The synthesis of the latter type of OPNOs often relies on the pre-assembly or self-assembly of polymeric substrates, which afford additional control over the structures and properties of OPNOs (Chen and Tseng, 2017; Jia et al., 2017). Because of the heterogeneous nature of their photoactive domains, many OPNOs absorb broadly from UV to near infrared (NIR) light, making them promising candidates as photocatalysts, due to the tunable irradiation wavelength (Zhu et al., 2015b).

Nonetheless, two main limitations remain for OPNO-based OPCs. First, most reports only focused on reactions in organic solvents. Water-soluble photocatalysts and aqueous photochemical reactions were largely omitted. Second, reactions using long-wavelength irradiations (other than blue or UV light) were underexplored, despite the advantages of long-wavelength irradiations, such as better penetration depths (Romero and Nicewicz, 2016; Han et al., 2018). Overcoming these limitations is necessary for expanding the applications of OPNO-based OPCs, obtaining deeper understandings of the photocatalytic mechanism, and economizing the photocatalytic processes (Zhang et al., 2020).

The key to solve these challenges is advancing the synthesis of OPNOs, which is traditionally limited to a narrow selection of substrates, such as citric acid/ethylenediamine (Zhu et al., 2013), conjugated polymers (Zhao et al., 2019) etc., Recently, polyacrylonitrile (PAN) has caught the attention of many researchers. PAN has been used as a substrate in the manufacturing of carbon fibers and heteroatom-doped nanocarbons (Tang et al., 2005; Zhong et al., 2012; Kopeć et al., 2017; Gottlieb et al., 2019; Kopeć et al., 2019; Yuan et al., 2019; Yuan et al., 2020). The photoluminescent properties of PAN were largely ignored, since untreated isolated nitrile groups do not form photoactive conjugated structures. However, recent reports point out that PAN can generate photoluminescence when dissolved at high concentrations or densely grafted from flat silica surfaces (Jang et al., 2006; Zhou et al., 2016; Kopeć et al., 2020). Additionally, there are recent reports of chemical conversion of PAN into photoactive species *via* microwave (Go et al., 2017), pyrolysis (Cao et al., 2020) and hydrothermal reactions (Ermakov et al., 2000; Sun et al., 2020), which all can induce the crosslinking or the hydrolysis of -C≡N, resulting in photoluminescent crosslinked nanoparticles or polymers. In particular, we recently reported the synthesis of visible light-absorbing photoluminescent polymers (PLPs) from PAN *via* a one-step hydrothermal reaction (Sun et al., 2020). In certain solvents (e.g., water), the resulting PLPs can self-assemble into polymer dot-like OPNOs. For example, the hydrothermally synthesized photoluminescent polymer (*ht*-PLP_PAN_) assembles in water into spherical aggregates with an average diameter of ca. 20 nm, due to the presence of both hydrophilic (i.e., carboxylic) and hydrophobic (i.e., aliphatic carbons) moieties. The assembly behavior was studied mainly by dynamic light scattering (DLS) in multiple solvents, including water and dimethyl sulfoxide (DMSO) (Sun et al., 2020). The high water solubility (>100 mg/ml) makes *ht*-PLP_PAN_ a promising candidate as an OPNO-based OPC in aqueous systems.

One of the most particularly promising photochemical processes in polymer chemistry is photoinduced controlled radical polymerization (CRP), which has emerged as a powerful and versatile method for controlled polymer syntheses (Pan et al., 2016a; Chen et al., 2016; Dadashi-Silab et al., 2016; Corrigan et al., 2020; Parkatzidis et al., 2020). Similar to other externally controlled CRP methods (Chmielarz et al., 2017; Mohapatra et al., 2017; Wang et al., 2017; Pan et al., 2018), photoinduced CRPs exhibit many advantages, such as excellent temporal control. In particular, photoinduced atom transfer radical polymerization (ATRP) (Wang and Matyjaszewski, 1995; Matyjaszewski and Xia, 2001; Matyjaszewski, 2012; Dadashi-Silab et al., 2014; Matyjaszewski and Tsarevsky, 2014; Theriot et al., 2016; Matyjaszewski, 2018; Lorandi and Matyjaszewski, 2020) and reversible addition-fragmentation chain-transfer (RAFT) polymerization (Xu et al., 2015; Pearson et al., 2016; Perrier, 2017; Allegrezza and Konkolewicz, 2021) have largely benefited from the development of OPCs, such as phenothiazine derivatives (Treat et al., 2014; Pan et al., 2016b; Theriot et al., 2016; Dadashi-Silab et al., 2021), eosin Y (Kutahya et al., 2016; El Achi et al., 2020) and halogenated xanthene dyes (Wu et al., 2019). Besides small-molecule OPCs, OPNOs have been applied in photoinduced CRP (Jiang et al., 2018; Kütahya et al., 2020; Hao et al., 2021; Kütahya et al., 2021). For example, heteroatom-doped carbon dots were applied in photoinduced energy/electron transfer RAFT (PET-RAFT) polymerization of (meth)acrylate monomers (Jiang et al., 2018). Doping S or P to the catalyst enabled successful PET-RAFT polymerization under red light. Additionally, photoinduced ATRP using low ppm (parts per million) loadings of Cu complexes was performed using carbon dots or polymer dots under blue light irradiation (Kütahya et al., 2020; Kütahya et al., 2021). In these systems, carbon dots reduced the Cu^II^ complex to the corresponding Cu^I^ complex, which activated the alkyl bromide initiator. Cu^II^ complexes functioned as deactivators to provide control over the polymerization (Kütahya et al., 2020).

Nevertheless, photoinduced CRPs in the presence of OPNOs primarily employed organic solvents and oleophilic monomers (Jiang et al., 2018; Kütahya et al., 2020; Kütahya et al., 2021). Although photoinduced CRPs in aqueous media were reported using other types of catalysts (Konkolewicz et al., 2012; Pan et al., 2015; Szczepaniak et al., 2020), extending these processes to new catalysts with different structures and photoluminescent mechanisms is needed to expand the understanding of the reaction mechanisms. Furthermore, ATRP using OPNOs under long-wavelength irradiations is underexplored compared to RAFT polymerization.

**SCHEME 1:**
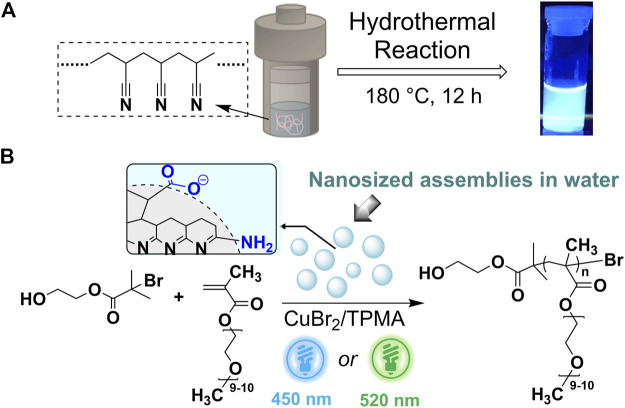
**(A)** Schematic representation of the synthesis of *ht*-PLP_PAN_, and an image of *ht*-PLP_PAN_ dissolved in water under UV light excitation (365 nm) **(B)** Proposed assembled structure of *ht*-PLP_PAN_ based on the previous report (Sun et al., 2020), and the use of *ht*-PLP_PAN_ as the photo-cocatalyst in Cu-catalyzed light-mediated ATRP. Functional groups highlighted in blue represent typical hydrophilic fragments.

Herein, we expand the applicability of photoinduced ATRP by developing Cu-catalyzed ATRP in the presence of *ht*-PLP_PAN_ as OPCs under blue (*λ*_max_ = 450 nm) and green (*λ*_max_ = 520 nm) light irradiation. During initial attempts, *ht*-PLP_PAN_ did not initiate the polymerization when used as a photoinitiator in free radical polymerization (FRP) under inert atmosphere (N_2_). Nonetheless, well-controlled polymerizations were successfully performed in water and in organic solvents (DMSO, dimethylformamide or DMF, and anisole) in the presence of alkyl bromide initiators and 25–500 ppm of Cu^II^ complexes (Br-Cu^II^/L, L = ligand). A low loading of *ht*-PLP_PAN_ (<1 mg/ml) and moderate light intensities (4–6 mW.cm^−2^) were used. Aqueous Cu-catalyzed ATRP of a water-soluble monomer, oligo (ethylene glycol) methyl ether methacrylate (OEGMA), is shown in Scheme 1. Studies performed under different irradiation wavelengths revealed that under blue light irradiation *ht*-PLP_PAN_ could both reduce the Cu^II^ complexes and activate alkyl halides. In contrast, under green light irradiation it was only capable to reduce the Cu^II^ complexes.

## Materials and Methods

### Materials

Methyl methacrylate (MMA, 99%, Sigma-Aldrich, United States), oligo (ethylene glycol) methyl ether methacrylate (OEGMA, average MW = 500, Sigma-Aldrich, United States), acrylonitrile (AN, 99%, Sigma-Aldrich) and methyl acrylate (MA, 99%, Sigma-Aldrich, United States) were purified by passing the monomers through a column filled with basic alumina to remove the inhibitor. Deionized water (DI water) was obtained from Millipore-Sigma Milli-Q water purification system. Azobisisobutyronitrile (AIBN, 98%, Sigma-Aldrich, United States) was recrystallized in anisole and stored at 4°C in dark. Tris (2-pyridylmethyl)amine (TPMA) was synthesized based on previously reported methods (Xia and Matyjaszewski, 1999). Copper bromide (CuBr_2_, 99%, Acros Organics, United States), ethyl α-bromoisobutyrate (EBiB, 98%, Sigma-Aldrich, United States), ethyl α-bromophenylacetate (EBPA, 97%, Sigma-Aldrich, United States), 2-hydroxyethyl 2-bromoisobutyrate (HO-EBiB, 95%, Sigma-Aldrich, United States), 2-bromopropionitrile (BPN, 97%, Sigma-Aldrich, United States), deuterated DMSO (DMSO-d_6_, 99.9%, Cambridge Isotope Laboratories, United States), deuterium oxide (D_2_O, 99.9%, Cambridge Isotope Laboratories), anisole (99%, Sigma-Aldrich, United States), dimethyl sulfoxide (DMSO, 99.7%, Fisher, United States), toluene (99%, Fisher, United States), and dimethylformamide (DMF, 99.8%, Fisher, United States) were used as received. Tetraethylammonium tetrafluoroborate (Et_4_NBF_4_, 99%, Alfa Aesar, United States), used as a supporting electrolyte for electrochemical analysis, was recrystallized from ethanol, and dried in a vacuum oven at 70°C for 48 h.

### Instruments

^1^H nuclear magnetic resonance (NMR) was conducted on a Bruker 500 MHz AVANCE III NMR spectrometer. Polymerizations in organic solvents used DMSO-d_6_ as the solvent for ^1^H NMR, and D_2_O for aqueous ATRP. DMF GPC was equipped with a refractive index (RI) detector, an Agilent 1260 Infinity II pump and a Wyatt Optilab T-rEX RI detector, with a PSS GRAM analytical column set (10 µm particle size) and LiBr-containing HPLC grade DMF as the eluent (LiBr: 0.05 M). Agilent EasiVial poly (methyl methacrylate) (PMMA) calibration kit (part number: PL 2020-0200) was used to calibrate the RI detector of GPC. Blue (*λ*_max_ = 450 nm, 5.0 mW.cm^−2^) and green (*λ*_max_ = 520 nm, 4.7 mW.cm^−2^) LED lights were purchased from aspectLED. The light intensity was measured by a ThorLabs PM100D compact power and energy meter console. The photoreactor for polymerization was prepared by mounting LED strips inside a round glass container, and air flow was applied to limit temperature increase during the polymerization. Solvent evaporation was performed on a Biotage V10 rapid solvent removal system. UV-vis spectra were taken on an Agilent Cary 60 UV-vis spectrometer and recorded by Cary WinUV software. Linear sweep voltammetries were measured by an Autolab PGSTAT302N potentiostat/galvanostat (Metrohm) run by a computer with NOVA 2.0 software. Dynamic light scattering (DLS) was measured by a Malvern Zetasizer Ultra light scattering system. Transmission electron microscopy (TEM) was performed using a JEOL JEM-2000EX TEM.

### Synthesis of *ht*-PLP_PAN_

*ht*-PLP_PAN_, was synthesized *via* previously reported procedures (Sun et al., 2020). PAN_165_ (subscript defines the degree of polymerization) was used to prepare *ht*-PLP_PAN_ and was synthesized by initiators for continuous activator regeneration (ICAR) ATRP using BPN as the initiator (Lamson et al., 2016; Kopeć et al., 2017). In a typical procedure, a 10 mg/ml suspension of ball-milled PAN_165_ in DI water was prepared. 10 ml suspension was stirred for 10 min and then transferred to a 25 ml autoclave reactor with high-temperature resistant liner. The autoclave reactor was securely sealed and placed in a pre-heated oven (180°C). The heating was turned off after 12 h and the oven was let to cool down to room temperature naturally. The dark brown solution was passed through a 0.22 µm syringe filter with a polyethersulfone membrane. The solution was directly dried by vacuum to yield brown solid *ht-*PLP_PAN_.

### General Procedure for Light-Mediated ATRP in the Presence of *ht*-PLP_PAN_

In a typical procedure, 5 mg *ht*-PLP_PAN_ was added to a 10 ml Schlenk flask containing 0.3 ml DMF and 7.2 ml DI water with a magnetic stir bar. HO-EBiB (3.9 µL, 0.027 mmol, 1 eq), CuBr_2_ (0.60 mg, 2.7 µmol, 0.1 eq), TPMA (2.35 mg, 8.1 µmol, 0.3 eq), NaBr (41.17 mg, 0.4 mmol), OEGMA (2.5 ml, 5.4 mmol, 200 eq) were subsequently added to the Schlenk flask. The Schlenk flask was then purged with N_2_ for approx. 25 min, and 0.1 ml of the reaction was withdrawn and was used as the “t = 0” sample. Finally, the Schlenk flask was placed in the photoreactor, and light was turned on to start the polymerization. The conversion of OEGMA was monitored by ^1^H NMR by withdrawing samples (∼0.1 ml each time) from the reaction mixture at different time points.

### General Procedures for Voltammetric Measurements

Linear sweep voltammetries were carried out in a 5-neck electrochemical cell placed inside the photoreactor, equipped with a 3-electrode system and connected to an Autolab PGSTAT302N potentiostat/galvanostat (Metrohm) controlled by NOVA 2.0 software. The 3-electrode system was composed by: 1) a Pt foil counter electrode; 2) a homemade quasi-reference electrode: Ag|AgI|(0.1 M *n*-Bu_4_NI in DMF); 3) a glassy carbon (GC) disk tip (3 mm dia, Metrohm), connected to a rotating disk electrode (RDE) system, as working electrode. Before each experiment, the GC disk was cleaned by polishing with a 0.25-μm diamond paste, followed by ultrasonic rinsing in ethanol for 5 min. Ferrocene (Fc) was added at the end of each experiment as an internal standard, to refer all potentials to the saturated calomel electrode [SCE, *E*°(Fc^+^|Fc) = 0.475 V vs SCE in DMF]. A steady air flow was applied to limit the temperature increase caused by light irradiation, and all experiments were performed under inert atmosphere (N_2_).

## Results and Discussion

### Photophysical Properties of *ht*-PLP_PAN_

*ht*-PLP_PAN_ was synthesized based on a previously reported hydrothermal reaction (Sun et al., 2020). Dynamic light scattering (DLS) measurement suggested that *ht*-PLP_PAN_ formed aggregates in water (Supplementary Figure 1). Transmission electron microscopy (TEM) revealed that the average diameter of the quasi-spherical assemblies was ca. 20 nm (Supplementary Figure 2). At first, using *ht*-PLP_PAN_ as a photoinitiator in FRP was attempted under blue or green light irradiation. However, no polymerization was observed under N_2_ atmosphere in the absence of additional initiators (Table 1, entries 5 and 9, discussed in detail later). Thus, our focus shifted to using *ht*-PLP_PAN_ as a reducing agent for the CuBr_2_ complex with a common ATRP ligand, tris(2-pyridylmethyl)amine (TPMA). (Xia and Matyjaszewski, 1999).

**TABLE 1:** Cu-catalyzed photoinduced ATRP of OEGMA_500_ in water^a^ with HO-EBiB as the initiator and *ht*-PLP_PAN_ as the photo-cocatalyst (0.5 mg/ml).

Entry	Irradiation	[M]_0_/[I]_0_/[CuBr_2_]_0_/[TPMA]_0_	Conv. (%)	*M*_n,theo_ ^b^	*M*_n,GPC_ ^c^	*Đ*
1	Blue (450 nm)^d^	200/1/0.1/0.3	98 (2.5 h)	98,300	50,700	1.35
2		200/1/0.02/0.06	92 (3 h)	92,000	72,600	1.69
3		200/1/0.02/0.06 (no *ht*-PLP_PAN_)	<5 (8 h)	–	–^e^	–
4		200/1/0/0	56 (8 h)	56,400	413,000	2.74
5		200/0/0/0	<5 (12 h)	–	–	–
6	Green (520 nm)^f^	200/1/0.1/0.3	87 (5 h)	87,700	49,100	1.38
7		200/1/0.02/0.06	81 (12 h)	81,900	62,200	1.55
8		200/1/0/0	<5 (48 h)	–	–	–
9		200/0/0/0	<5 (20 h)	–	–	–

a General conditions: Vol%(OEGMA) = 25%, and H_2_O (containing 40 mM NaBr and 3 vol% of DMF as internal standards for ^1^H NMR) was used as the solvent.

b M_n,theo_ was determined by the monomer conversion monitored by ^1^H NMR.

c M_n,GPC_ was calculated from a linear PMMA calibration.

d Intensity: 5.0 mW cm^−2^.

e No polymer signal was observed from GPC.

f Intensity: 4.7 mW cm^−2^.

**FIGURE 1:**
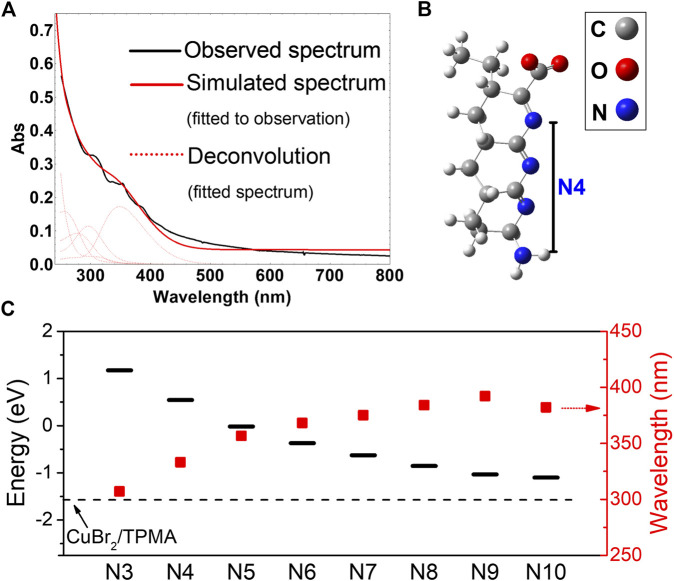
Photophysical properties of *ht*-PLP_PAN_ **(A)** Experimental and simulated UV-vis spectra of *ht*-PLP_PAN_ calculated by time-dependent density functional theory (TDDFT). Structure optimization and TDDFT calculations were performed in simulated water environment using a polarizable continuum model, at the ωB97X-D/6-31g-d and cam-b3lyp/6-31g-d levels, respectively **(B)** A representative model structure for photoactive domains of *ht*-PLP_PAN_ with four nitrogen atoms (*o*I_4_), optimized at the ωB97X-D/6-31g-d level (Stephens et al., 1994; Frisch et al., 2016). **(C)** Energy levels of the lowest unoccupied molecular orbital (LUMO, black bars) and wavelengths of the lowest energy transition (red squares) of model structures (*o*I_3_ to *o*I_10_) with different conjugation lengths represented by the number of nitrogen atoms within the backbone (Sun et al., 2020). The dashed line represents the energy of the LUMO (*β*) of the [Br-Cu^II^/TPMA]^+^ complex in water [optimized at the ωB97X-D level with def2TZVP basis set on Cu and 6-31g-d basis set on the other atoms (Miertuš and Tomasi, 1981; Chai and Head-Gordon, 2008)].

To evaluate the capability of *ht*-PLP_PAN_ in photoreducing the CuBr_2_/TPMA complex, photophysical properties of *ht*-PLP_PAN_ (Figure 1) were analyzed. Our previous study reported a broad UV-vis absorption profile (Figure 1A) and a short lifetime of the excited state *ht*-PLP_PAN_ (<5 ns), suggesting the singlet nature (Sun et al., 2020). Putative photophysical properties of *ht*-PLP_PAN_ were modeled using density functional theory (DFT) calculations with model oligoimine-based structures of different conjugation lengths *o*I_N_ (where N = 3–10 denotes the number of nitrogen atoms along the backbone), which were deemed likely to arise in the course of hydrothermal treatment (Figure 1B, see Supplementary Material for Cartesian coordinates). (Sun et al., 2020) In all instances, the energies of the lowest unoccupied molecular orbital (LUMO) of *o*I_N_ were higher than the LUMO (*β*) of **[**Br-Cu^II^/TPMA**]** ^**+**^, indicating conditions favorable for reduction of the Cu^II^ complex upon photoexcitation (Figure 1C). The LUMO_*o*IN-_LUMO_**[**Br-Cu_^II^_/TPMA**]**_^**+**^ gap increased, and the wavelength of the lowest-energy transition determined by time-dependent DFT (TDDFT) decreased with the decrease of N (Supplementary Figure 3), suggesting stronger “reducing power” of blue light absorbing species, in agreement with experimental observations. Experimental studies on the photoreduction of the Cu^II^ complex were performed using linear sweep voltammetry (LSV), which are discussed in *Photocatalytic Mechanism and Comparison Between Activation by Cu* ^*I*^ *Complex and by ht-PLP*_*PAN*_ section.

### Cu-Catalyzed ATRP in Water Using *ht*-PLP_PAN_

Blue (*λ*_max_ = 450 nm) and green light sources (*λ*_max_ = 520 nm) were chosen for photoinduced ATRP using *ht*-PLP_PAN_. Oligo (ethylene glycol) methyl ether methacrylate with an average molecular weight of 500 (OEGMA_500_) was polymerized using ATRP catalyzed by a CuBr_2_-complex with TPMA as the ligand (molar ratio: CuBr_2_/TPMA = 1/3) (Table 1). 100 ppm or 500 ppm (relative to the monomer concentration) of CuBr_2_/TPMA were used, with 2-hydroxyethyl 2-bromoisobutyrate (HO-EBiB) as the ATRP initiator and 0.5 mg/ml *ht*-PLP_PAN_ as the photo-cocatalyst. Additionally, 40 mM of NaBr was added to suppress the dissociation of the weak Cu^II^-Br bond in the ATRP deactivator (Simakova et al., 2012; Fu et al., 2018).

**FIGURE 2:**
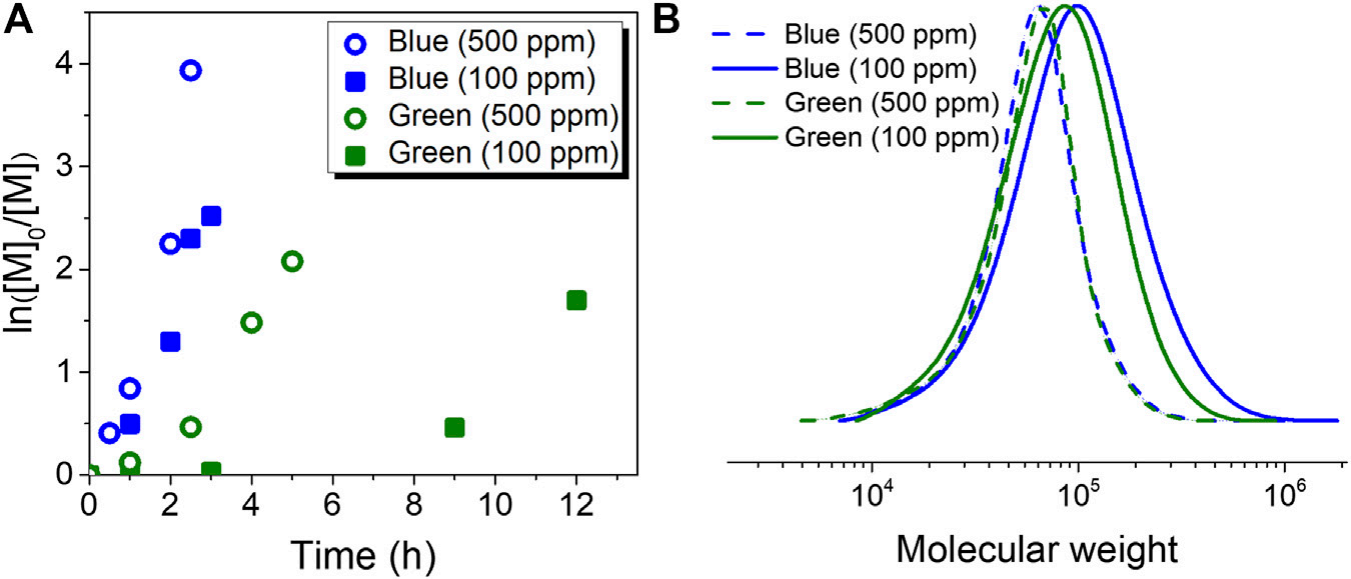
Photoinduced ATRP in water **(A)** First-order kinetic plots corresponding to entries 1 (blue, 500 ppm), 2 (blue, 100 ppm), 6 (green, 500 ppm) and 7 (green, 100 ppm) of Table 1 **(B)** GPC traces corresponding to the polymer product of entries 1, 2, 6 and 7 of Table 1.

First, polymerizations were performed under blue light irradiation. Linear semilogarithmic kinetic plots for ATRP using both 500 ppm (entry 1, Table 1) and 100 ppm (entry 2, Table 1) of the Cu^II^ complex are shown in Figure 2A. Polymer molecular weight and dispersity (*Đ*) were measured by gel permeation chromatography (GPC) using dimethylformamide (DMF) as the mobile phase (Figure 2B). Increasing the loading of CuBr_2_/TPMA from 100 ppm (entry 2) to 500 ppm (entry 1) resulted in a decreased *Đ* from 1.69 (entry 2) to 1.35 (entry 1), due to the higher equilibrium concentration of the deactivator. Clean shifts of the molecular weight distribution (MWD) traces are shown in Supplementary Figure 4. It is worth mentioning that the difference in the molecular weight measured from the light scattering (LS) detector of GPC (*M*_n,GPC_) and the theoretical molecular weight (*M*_n,theo_) was likely due to different polymer-column interactions between poly (OEGMA) and the calibration standard (PMMA) used for the calibration of the LS detector. Figure 2A also illustrates that the rate of polymerization for entry 1 (500 ppm of Cu complex) was faster than that for entry 2 (100 ppm of Cu complex). This difference was attributed to the higher concentration of propagating radicals in entry 1 and faster reduction of the Cu^II^ complex. Similar kinetic results were previously reported by several ATRP methods based on activator regeneration (Simakova et al., 2012; Mendonça et al., 2014). Despite the lower monomer conversion determined by ^1^H NMR, entry 2 showed a higher *M*_n,GPC_ than entry 1 (Table 1). This difference indicated that the initiation efficiency of HO-EBiB was lower when a lower loading of CuBr_2_/TPMA was used in entry 1.

In order to confirm the role of *ht*-PLP_PAN_, a control experiment was performed in its absence (entry 3, Table 1). No substantial monomer conversion (<5% after 8 h) was observed, indicating that the *ht*-PLP_PAN_ was required to generate the Cu^I^ activator complex. In addition, the possibility of direct activation of the alkyl bromide (R-Br) initiator by *ht*-PLP_PAN_ was considered. A control experiment (entry 4, Table 1) showed that under blue light irradiation HO-EBiB was indeed activated in the presence of *ht*-PLP_PAN_ and in the absence of the Cu^II^ complex. The polymerization was uncontrolled (*Đ* = 2.74) due to the absence of Cu^II^ deactivators. In comparison, no polymerization occurred when no HO-EBiB was added, indicating that *ht*-PLP_PAN_ did not generate radicals directly from the monomer. A quantitative comparison between the activation of R-Br by the Cu^I^ complex and by *ht*-PLP_PAN_ is presented in the *Photocatalytic Mechanism* section.

Similar polymerizations were conducted under green light irradiation (entries 6–7, Table 1 and Figure 2). In general, polymerization rates under green light irradiation were slower than those under blue light irradiation, and proceeded only after an induction period (Figure 2A). Finally, similar control experiments (entries 8 and 9, Table 1) were performed under green light. In contrast with the control experiments performed under blue light, no polymerization was observed under green light irradiation in the absence of Cu^II^, indicating that *ht*-PLP_PAN_ luminophores excited by this range of wavelengths were not capable to activate the alkyl bromide initiator under green light, even after prolonged irradiation (48 h, entry 8, Table 1).

In addition to the already discussed lower “reducing power” of longer-wavelength luminophores inferred from DFT model calculations, two other factors that could be responsible for the slower polymerization and absence of *ht*-PLP_PAN_-driven activation of R-Br under green light are: 1) the lower light absorption of *ht*-PLP_PAN_ at 520 nm (Figure 1), and 2) the slightly lower intensity of the green light photoreactor (4.7 mW.cm^−2^, vs 5.0 mW.cm^−2^ of the blue light photoreactor). Nevertheless, green light irradiation still resulted in reasonably well-controlled polymerization of OEGMA_500_ (*Đ* = 1.38) using 500 ppm of Cu^II^/TPMA (entry 6, Table 1).

### Cu-Catalyzed ATRP in Organic Solvents Using *ht*-PLP_PAN_

To demonstrate the versatility of *ht*-PLP_PAN_ as OPCs, a mixed solvent containing 1:1 (v/v) of DMSO and DMF was first used to polymerize MMA with ethyl α-bromophenylacetate (EBPA) as the initiator. It is worth mentioning that *ht*-PLP_PAN_ only partially dissolved when using DMSO/DMF due to the lower polarity of the reaction media, especially after the addition of MMA. Despite the weaker solubility of *ht*-PLP_PAN_, well-controlled polymerizations were obtained with *ht*-PLP_PAN_ dispersed in the solvent using 100 ppm or 25 ppm of CuBr_2_/TPMA as the catalyst (Table 2).

**TABLE 2:** Cu-catalyzed light-mediated ATRP of MMA in DMSO/DMF^a^ with EBPA as the initiator and *ht*-PLP_PAN_ as the photo-cocatalyst (0.95 mg/ml).

Entry	Irradiation	[M]_0_/[I]_0_/[CuBr_2_]_0_/[TPMA]_0_	Conv. (%)	*M*_n,theo_ ^b^	*M*_n,GPC_ ^c^	*Đ*
1	Blue (450 nm)^d^	200/1/0.02/0.06	70 (28 h)	14,200	11,900	1.19
2		200/1/0.005/0.015	63 (25 h)	12,900	9,900	1.60
3		200/1/0/0	49 (12 h)	10,100	120,000	1.92
4		200/0/0/0	<5 (15 h)	–^e^	–	–
5	Green (520 nm)^f^	200/1/0.02/0.06	66 (24 h)	13,400	13,800	1.30
6		200/1/0.005/0.015	40 (25 h)	8,400	8,300	1.41
7		200/1/0/0	<5 (12 h)	–	–	–

a General conditions: Vol%(MMA) = 25% and DMSO/DMF (vol ratio = 1/1) was used as the solvent. M_n,GPC_ was calculated from PMMA calibrations.

b M_n,theo_ was determined by the monomer conversion monitored by ^1^H NMR.

c M_n,GPC_ was calculated from a linear PMMA calibration.

d Intensity: 5.0 mW cm^−2^.

e No polymerization was observed on GPC.

f Intensity: 4.7 mW cm^−2^.

**FIGURE 3:**
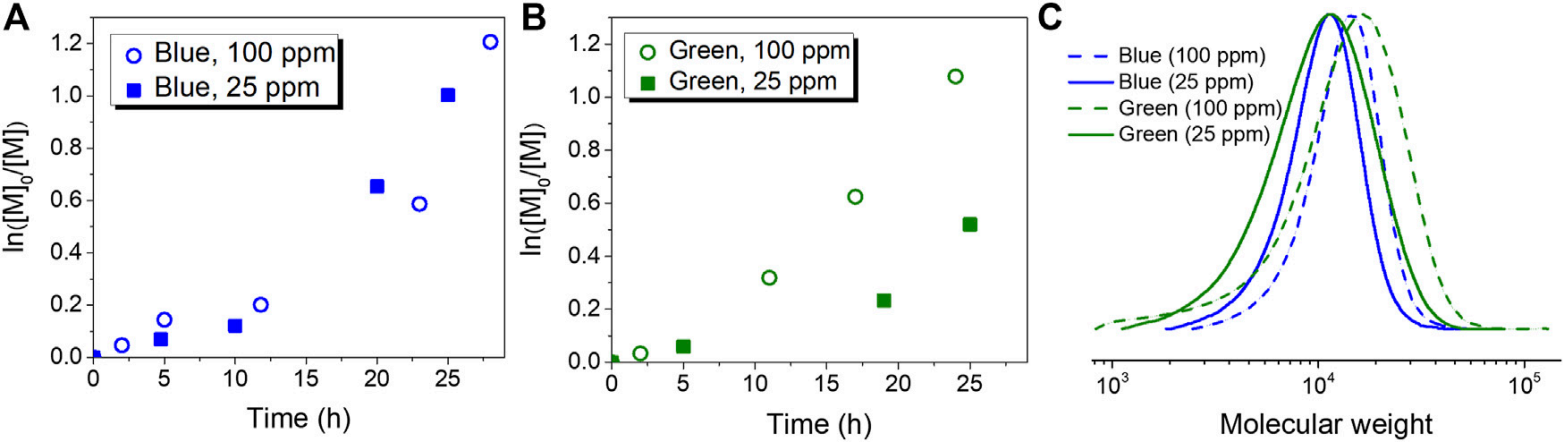
Photoinduced ATRP in organic solvents. First-order kinetic plots corresponding to **(A)** entries 1 (blue, 100 ppm) and 2 (blue, 25 ppm) **(B)** entries 5 (green, 100 ppm) and 6 (green, 25 ppm) of Table 2. **(C)** GPC traces corresponding to the polymer product of entries 1, 2, 5 and 6 of Table 2.

Similar to the case of aqueous media, better control was obtained for the polymerization of MMA with a higher loading of CuBr_2_/TPMA (entries 1 and 5, Table 2). However, 500 ppm of the Cu complex were required to control the process in aqueous media (entries 1 and 6, Table 1), while concentrations of Cu^II^ complex as low as 25 ppm were sufficient in organic solvents (entry 6, Table 2). Linear semilogarithmic kinetic plots and the GPC traces observed under those conditions are shown in Figure 3. Clean shifts of GPC traces were also observed (Supplementary Figure 5). Additionally, the direct activation of EBPA by *ht*-PLP_PAN_ was evaluated by control experiments in the absence of CuBr_2_/TPMA (entries 3 and 7, Table 1). Similar to results in aqueous media, *ht*-PLP_PAN_ activated EBPA only under blue light irradiation. The supplemental activation of EBPA under blue light irradiation likely contributed to the higher dispersity when 25 ppm loading of CuBr_2_/TPMA was used (entry 2). The uncontrolled polymerization in the absence of any Cu complexes indicated that no deactivations occurred due to the absence of deactivators.

Finally, ATRP using *ht*-PLP_PAN_ was extended to other monomers and solvents (Supplementary Table 1). For example, methyl acrylate (MA) was polymerized using 100 ppm of the CuBr_2_/TPMA complex and ethyl α-bromoisobutyrate (EBiB) as the initiator (entry 1, Supplementary Table 1). Good control over the polymerization was illustrated by the low *Đ* = 1.16 of the polymer (Supplementary Figures 6A,B). Additionally, ATRP of MMA was performed using 100 ppm of the CuBr_2_/TPMA complex and anisole as a less polar solvent (entry 2, Supplementary Table 1). The rate of polymerization was slower in anisole compared to DMSO/DMF, as expected from the variation of the ATRP equilibrium constant, *K*_ATRP_, with solvent polarity (Ribelli et al., 2019). Nonetheless, well-controlled polymerization (*Đ* = 1.19) was still observed (Supplementary Figures 6C,D).

### Photocatalytic Mechanism and Comparison Between Activation by Cu^I^ Complex and by *ht*-PLP_PAN_

**FIGURE 4:**
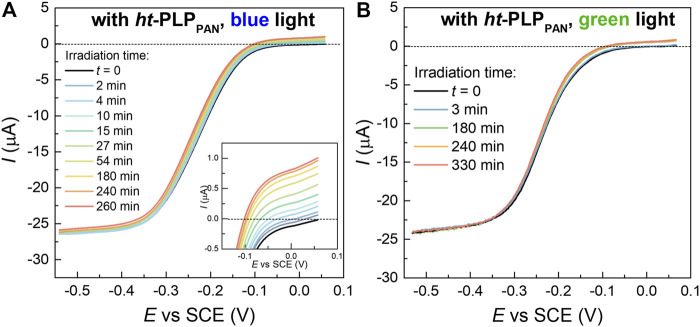
LSV of 0.6 mM CuBr_2_/TPMA (L/Cu = 3) in DMSO-DMF (volume ratio: 1/1) + 0.1 M Et_4_NBF_4_ at *v* = 0.01 V.s^−1^ and RDE rotation speed 4,000 rpm, under **(A)** blue light and **(B)** green light irradiation, in the presence of *ht*-PLP_PAN_ (1.5 mg/ml).

In some ATRP methods with regeneration of the Cu^I^/L activator, the agent used for the activator regeneration can also contribute to the activation of dormant alkyl bromides to form propagating radicals, which generally occurs by reaction between the agent and the alkyl bromide (Konkolewicz et al., 2013; Konkolewicz et al., 2014a; Konkolewicz et al., 2014b; Zhang et al., 2011). To better understand the polymerization mechanism of the current system, the photoreduction of the Cu^II^ complex by *ht*-PLP_PAN_ was first monitored by linear sweep voltammetry (LSV). As shown in Figure 4, LSV was scanned from ca. 0.5 V (vs SCE) where the Cu^I^ complex is oxidized if it exists. Indeed, the increased intensity of the anodic current under blue or green light irradiation confirmed the formation of Cu^I^/TPMA complex in DMSO/DMF (volume ratio: 1/1) (Dadashi-Silab et al., 2021). The role of *ht*-PLP_PAN_ as the primary factor behind the observed reduction of Cu^II^/TPMA complexes after 3 h of blue light irradiation was confirmed by comparing the fraction of Cu^I^ complexes estimated from the current values in the presence of *ht*-PLP_PAN_ (ca. 2.5%) and in its absence (<0.5%, Supplementary Figure 7A). The small extent of photoreduction in the latter case was likely caused by the presence of excess ligand that can act as the electron donor. Notably, the LSV traces acquired in water did not show a consistent increase of Cu^I^/L concentration (Supplementary Figure 7B), in contrast with polymerization results (Table 1) that indicated *ht*-PLP_PAN_ was capable of photoreducing the Cu^II^ complex in water. This apparent discrepancy indicates that the amount of Cu^I^ produced by *ht*-PLP_PAN_ in water was below the reliable LSV detectability threshold but was still sufficient to effectively initiate polymerization owing to the higher *K*_ATRP_ of Cu catalysts in water (Tang et al., 2008; Fantin et al., 2015). It should be pointed out that the apparent lower reducing efficiency of *ht*-PLP_PAN_ in water in comparison with organic solvents could be caused by its nano-assembly, which, while important for allowing these mostly hydrophobic species to function in water, would inevitably decrease the number of accessible photocatalytic sites.

To further understand the composition of the catalytic system for aqueous ATRP under blue light irradiation, the following calculations were performed to quantify the concentrations of Cu^I^ and Cu^II^ species in polymerizations corresponding to conditions in Table 1. The activation rates of alkyl bromides by Cu^I^/L and *ht*-PLP_PAN_ were estimated for the polymerization of OEGMA (entries 1, 2 and 4 of Table 1). First, the rate of activation by the Cu^I^ complex ($R_{\text{a}1}$) was given by Eq. (1), (Matyjaszewski 2012; Krys et al. 2016; Krys and Matyjaszewski 2017) thus the concentration of the Cu^I^ complex ($\left[{\text{Cu}}^{\text{I}}/\text{L}\right]$) was calculated. Eq. 2 shows the relationship between $\left[{\text{Cu}}^{\text{I}}/\text{L}\right]$ and the rate of polymerization, $R_{\text{p}}$:

$$R_{\text{a}1}=k_{\text{a}1}\left[{\text{Cu}}^{\text{I}}/\text{L}\right]\left[\text{RX}\right]$$ (1)

$$R_{\text{p}}=k_{\text{p}}\;K_{\text{ATRP}}\frac{\left[\text{RX}\right]\left[{\text{Cu}}^{\text{I}}/\text{L}\right]}{\left[\text{Br}-{\text{Cu}}^{\text{II}}/\text{L}\right]}\left[\text{M}\right]$$ (2)

where, [RX], [M], [Cu^I^/L] and [Br-Cu^II^/L] correspond to the concentration of the alkyl bromide initiator, monomer, Cu^I^ complex and Cu^II^ complex, respectively. $k_{\text{a}1}$ is the activation rate constant of HO-EBiB by Cu^I^/TPMA. $K_{\text{ATRP}}$ is the ATRP equilibrium constant for Cu^I^/TPMA with HO-EBiB, and $k_{\text{p}}$ is the rate coefficient of propagation for OEGMA. (Smolne et al., 2016)

On the other hand, the activation rate of alkyl bromides by *ht*-PLP_PAN_ ($R_{\text{a}-\text{pc}}$) was determined from Eq. 3:

$$R_{\text{p},\text{ without Cu}}=k_{\text{p}}\left[\text{M}\right]\left[\text{R}\right]=k_{\text{p}}\left[\text{M}\right]\sqrt{\frac{R_{\text{a}-\text{pc}}}{k_{\text{t}}}}$$ (3)

where, $R_{\text{p},\text{ without Cu}}$ refers to the rate of polymerization in the absence of Cu^II^ complex. $k_{\text{t}}$ is the termination rate constant of OEGMA, (Smolne et al., 2016) and [R·] is the concentration of propagating radicals.

Details of the calculation, including the scaling of kinetic parameters, are included in the Supplementary Material (Supplementary Tables 2–4) (Fantin et al., 2015; Fantin et al., 2017). Specifically, $R_{\text{p},\text{ without Cu}}$ was monitored by ^1^H NMR (entry 4 of Table 1, Supplementary Figure 8). The polymerization was slower when no Cu complex was present, and polymers with high molecular weight (over seven times higher than *M*_n,theo_) were observed by GPC, indicating a low initiation efficiency when *ht*-PLP_PAN_ was the only activator.

As shown in Table 3, the calculated $R_{\text{p},\text{ without Cu}}$ was 6.34 × 10^−6^ M.s^−1^, which was ca. one order of magnitude smaller than $R_{\text{p}}$[$R_{\text{p}}$ (100 ppm) = 5.46 × 10^−5^ M.s^−1^, $R_{\text{p}}$ (500 ppm) = 7.22 × 10^−5^ M.s^−1^]. Based on these values, the calculated $\left[{\text{Cu}}^{\text{I}}/\text{L}\right]$ was 5.5 × 10^−7^ M for ATRP using 100 ppm of CuBr_2_/TPMA, and 3.6 × 10^−6^ M for ATRP using 500 ppm of CuBr_2_/TPMA. Although Cu^I^/L only consisted of 1.0% (entry 1, Table 3) and 1.4 (entry 2, Table 3) of the total Cu species, the calculated values of $R_{\text{a}1}$ (Table 3) were five to six orders of magnitude higher than $R_{\text{a}-\text{pc}}$ (9.3 × 10^−10^ M.s^−1^). This significant difference in the activation rate was due to the high $k_{\text{a}1}$ value of [Cu^I^/TPMA]^+^ in aqueous media (Fantin et al., 2017), making Cu^I^/L the predominant activator over *ht*-PLP_PAN_ in this system.

**TABLE 3:** Calculated reaction parameters during light-mediated ATRP of OEGMA catalyzed by CuBr_2_/TPMA. Total concentrations for Cu species were 0.054 mM (entry 1, Table 1) and 0.27 mM (entry 2, Table 1), respectively. The concentration of HO-EBiB was 2.7 mM in both entries.

Entry	Loading of Cu complex (ppm)	*R*_p_ ^a^	[Cu^I^/L]	Cu^I^/L %^b^ (%)	*R*_a1_ ^c^
1	100	5.46 × 10^−5^ M.s^−1^	5.5 × 10^−7^ M	1.0	8.1 × 10^−5^ M.s^−1^
2	500	7.22 × 10^−5^ M.s^−1^	3.6 × 10^−6^ M	1.4	5.3 × 10^−4^ M.s^−1^

a Rate of polymerization.

b Molar fraction of Cu^I^/L complex during the polymerization.

c Activation rate of R-Br by Cu^I^/L complex. In comparison, the estimated activation rate of R-Br by *ht*-PLP_PAN_ (in the absence of Cu species) was 9.3 × 10^−10^ M.s^−1^. See Supplementary Material for calculation methods.

## Conclusions and Perspectives

In conclusion, a dispersible PAN-derived photo-cocatalyst, *ht*-PLP_PAN_, was applied as OPCs in Cu-catalyzed ATRP under blue or green light irradiation. Hydrophilic and hydrophobic functional groups prompted *ht*-PLP_PAN_ to assemble into spherical OPNOs in water. Compared to previously reported photoinduced CRPs using self-assembled photocatalysts, this work expanded the utilization of OPNO-based photocatalysts or photo-cocatalysts from organic solvents (DMF/DMSO, anisole) to aqueous media. Well-controlled polymerization of acrylate and methacrylate monomers were reported. Furthermore, analysis of the R-Br activation demonstrated how the irradiation wavelength affected the activation mechanism of Cu-catalyzed ATRP. Control experiments showed that both *ht*-PLP_PAN_ and the Cu^I^ complex could activate R-Br under blue light irradiation. While under green light irradiation, the Cu^I^ complex was the only activator for R-Br. But the reaction between the Cu^I^ complex and R-Br was five to six orders of magnitude faster than the activation of R-Br by *ht*-PLP_PAN_, due to the high activation rate constant of the Cu^I^ complex, particularly in aqueous media.

## Data Availability

The original contributions presented in the study are included in the article/Supplementary Material, further inquiries can be directed to the corresponding author.
