# Supplementary material for: Assemblies of Polyacrylonitrile-Derived Photoactive Polymers as Blue and Green Light Photo-Cocatalysts for Cu-Catalyzed ATRP in Water and Organic Solvents
Source: Front Chem. 2021 Aug 13;9:734076. doi: 10.3389/fchem.2021.734076 (PMC8407075; doi:10.3389/fchem.2021.734076)
Supplement: Supplementary file 1 [file DataSheet1.DOCX]

*Supporting Information for*

Assemblies of Polyacrylonitrile-Derived Photoactive Polymers as Blue and Green Light Photo-Cocatalysts for Cu-Catalyzed ATRP in Water and Organic Solvents

Mingkang Sun^1^, Francesca Lorandi^1^, Rui Yuan^1^, Sajjad Dadashi-Silab^1^, Tomasz Kowalewski^1^, Krzysztof Matyjaszewski^1*^

^1^Department of Chemistry, Carnegie Mellon University, Pittsburgh, Pennsylvania 15213, United States

*** Correspondence**Corresponding Author: matyjaszewski@cmu.edu

Contents

[1. Additional Procedures and Analysis 4](#_Toc75907597)

[1.1 Synthesis of PAN_165_ 4](#_Toc75907598)

[1.2 Light-mediated ATRP in organic solvents 4](#_Toc75907599)

[1.4 Procedures for the scaling of kinetic parameters 5](#_Toc75907600)

[2. Supplemental Figures and Tables 7](#_Toc75907601)

[3. DFT Calculations and Cartesian Coordinates 13](#_Toc75907604)

[REFERENCES 22](#_Toc75907605)

# 1. Additional Procedures and Analysis

## 1.1 Synthesis of PAN_165_

The synthesis of PAN_165_ was based on previously reported procedures.^1, 2^ 20 mL of AN (0.31 mol, 500 eq), 6.9 mg CuBr_2_ (0.031 mmol, 0.05 eq) and 26.8 mg TPMA (0.092 mmol, 0.15 eq) were charged into a Schlenk flask containing 25 mL of DMSO and 2.5 mL of DMF (^1^H NMR internal standard), and was purged with N_2_ for 20 min. 53.1 µL BPN (0.61 mmol, 1 eq) was then injected and the mixture was further degassed for 10 min. Finally, 10.1 mg of AIBN (0.061 mmol, 0.1 eq) was added and the Schlenk flask was quickly immersed in an oil bath that was pre-heated to 65 ºC to start the polymerization. 0.2 mL reaction mixture was withdrawn from the reaction vessel using N_2_-purged syringe and needle to measure the polymerization conversion via ^1^H NMR. The reaction was quenched at 6 h by exposure to air. After stirring in air for 1 h, the mixture was precipitated in 1 L MeOH and the filtered solid was dried in vacuum at room temperature overnight to give dry linear PAN.

## 1.2 Light-mediated ATRP in organic solvents

In a typical procedure (25 ppm of CuBr_2_/TPMA, DMSO/DMF), 5 mg *ht*-PLP_PAN_ was added to a 10 mL Schlenk flask containing 1.97 mL DMF and 1.97 mL DMSO with a magnetic stir bar. 10.8 µL EBPA (0.062 mmol, 1 eq), 0.069 mg CuBr_2_ (0.31 µmol, 0.005 eq), 0.27 mg TPMA (0.93 µmol, 0.015 eq), 1.31 mL MMA (12.3 mmol, 200 eq) were subsequently added to the Schlenk flask. The Schlenk flask was then purged with N_2_ for approx. 25 min, and 0.1 mL of the reaction was withdrawn and was used as the “t=0” sample. Finally, the Schlenk flask was placed in the photoreactor, and light was turned on to start the polymerization. The conversion of MMA was monitored by ^1^H NMR by withdrawing samples (~ 0.1 mL each time) from the reaction mixture at different time points. DMSO-d_6_ was used as the solvent for ^1^H NMR tests.

## 1.4 Procedures for the scaling of kinetic parameters

The following section describes the adjustment of kinetic parameters used for the quantification of the activation of alkyl bromides by the Cu^I^ complex vs. by *ht*-PLP_PAN_ under blue light irradiation in water (containing 40 mM of NaBr to enhance of stability of the deactivator).

***Calculation of k_p_, k_t_ of OEGMA***

According to ref [3]^3^, The *k*_p_ value of OEGMA (average MW=500) can be calculated from eq S1:

$ln(k_{p}/(M^{-1} s^{-1})=16.58-\frac{2.1\times{10}^{4}}{8.3144\times T/K}-1.86\times{10}^{-2}\times\frac{c_{OGEMA}}{wt\%}$ (S1)

Where, *T* is the reaction temperature, and $c_{OGEMA}$is the weight fraction of OEGMA_500_ in the polymerization mixture.

Based on this equation, the calculated *k*_p_ (OEGMA_500_) = 2.01 × 10^3^ M^-1^ s^-1^.

Additionally, the estimated *k*_t_ (OEGMA_500_) = 2.74 × 10^7^ M^-1^ s^-1^ based on the same reference.

***Scaling of K_ATRP_***

First, the estimated *K*_ATRP_ value of [Cu^I^/TPMA]/HO-EBiB in pure water was 0.18.^4^

Then, the effect of adding OEGMA monomer in water was considered. According to ref [5] (entries 7 and 13, Table 2)^5^, *k*_act_ of alkyl bromides by Cu^I^/TPMA in water containing 18 wt% (or 17 vol%) oligo(ethylene glycol) methyl ether acrylate (OEGA) is ca. 2 orders of magnitude smaller than *k*_act_ in pure water.

Assuming the decrease of *K*_ATRP_ from pure water to water containing 26 vol% OEGMA is similar to the decrease of *k*_act_, the scaled *K*_ATRP_ for the polymerization condition is 1.8×10^-3^.

***Scaling of k_a1_***

The activation rate constant (*k*_a1_) of [Cu^I^/TPMA]/HO-EBiB in pure water was 5.4 × 10^6^ M^-1^ s^-1^ (HO-EBiB was the best structure to mimic OEGMA that has *k*_a1_ available).^5^ Similar to the adjustment of *K*_ATRP_, *k*_a1_ of [Cu^I^/TPMA]/HO-EBiB in OEGMA/water mixture was scaled by multiplying 5.4 × 10^6^ M^-1^ s^-1^ by 0.01. Thus, the adjusted *k*_a1_ is 5.4 × 10^4^ M^-1^ s^-1^. Table S2 summarizes the scaled rate constants and equilibrium constants used for the quantification:

# 2. Supplemental Figures and Tables


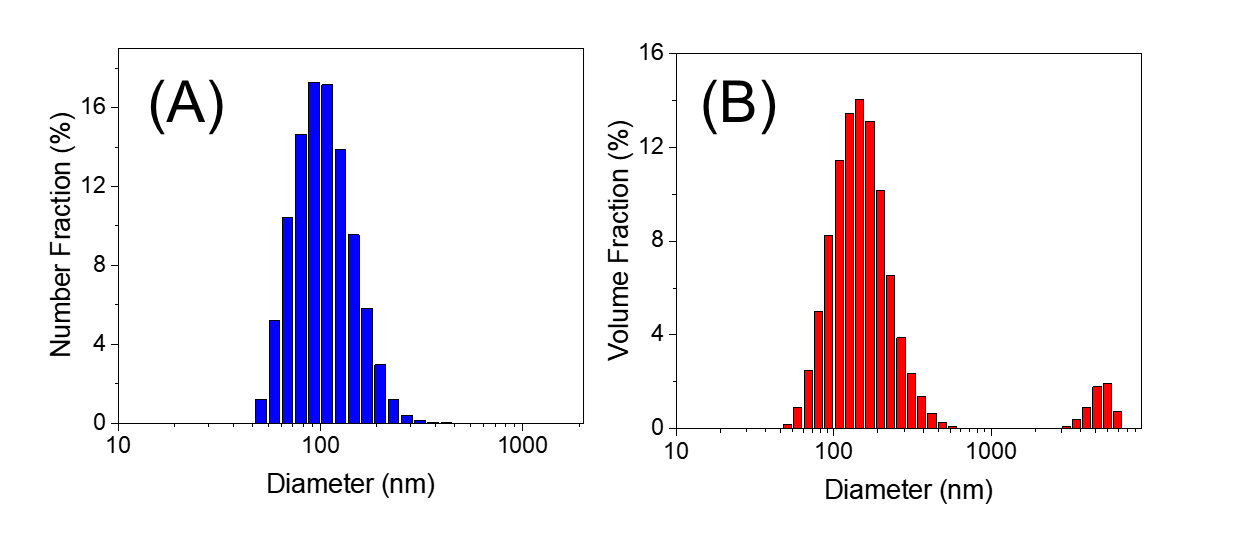


**Figure S1**. DLS (A) number fraction and (B) volume fraction distribution of *ht*-PLP_PAN_ in water (concentration of *ht*-PLP_PAN_: 0.5 mg/mL).


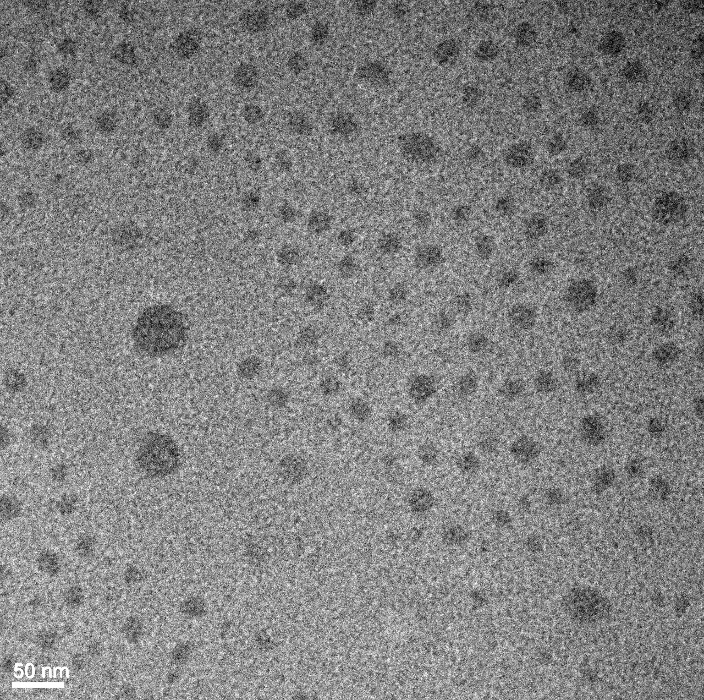


**Figure S2**. TEM image of a water dispersion of *ht*-PLP_PAN_ on a carbon film supported by a Cu grid. The TEM sample was prepared by drop-casting a water solution of *ht*-PLP_PAN_ (concentration: 1 mg/mL) onto the carbon film and dried in air for 2 h.

**Figure S3**. Simulated UV-vis spectra with transitions scaled to the oscillator strength of ***o*I_3_** to ***o*I_10_**, optimized at ωB97X-D/6-31g-d level with PCM water solvation model, and TD-DFT was calculated at cam-b3lyp/6-31g-d level.


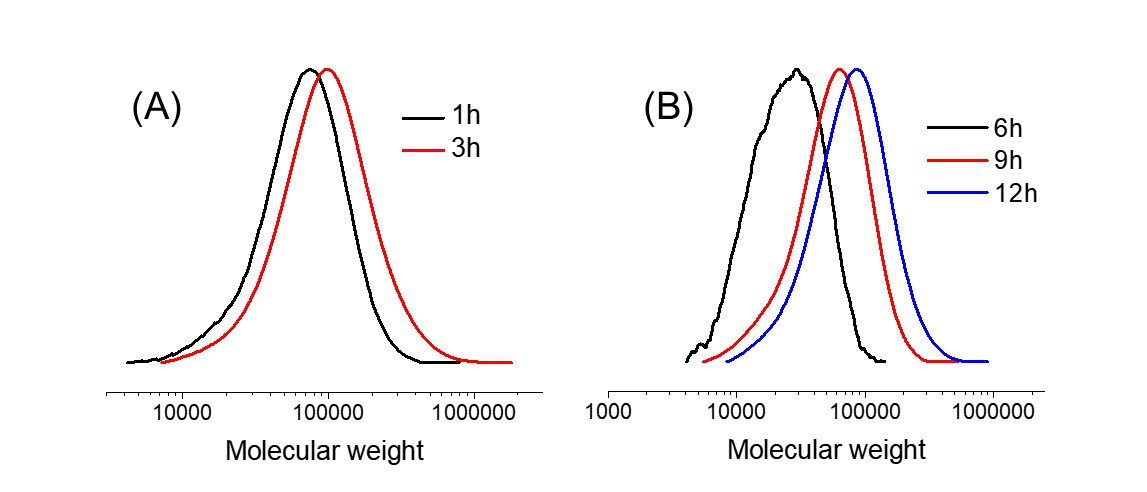


**Figure S4**. Evolution of GPC traces of (A) entry 2 and (B) entry 7 of Table 1 at different reaction time.


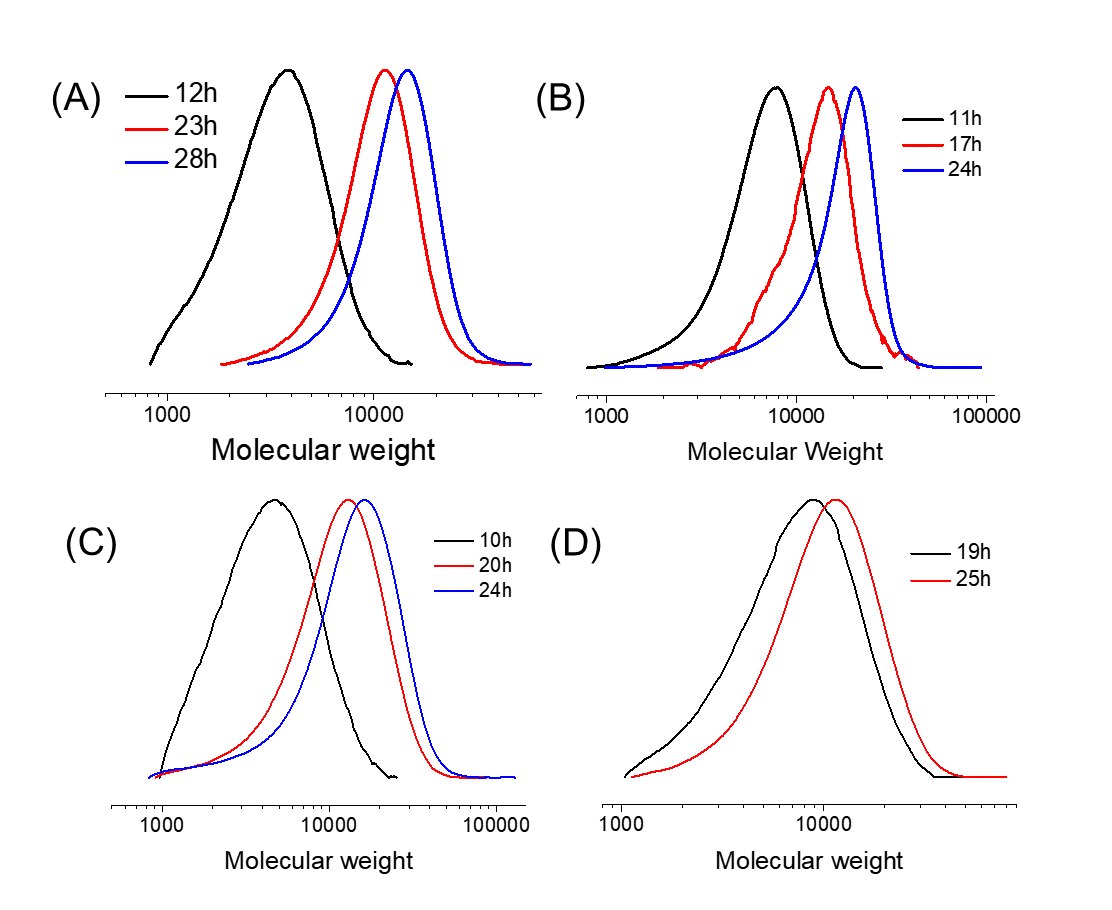


**Figure S5**. Evolution of GPC traces corresponding to (A) entry 1, (B) entry 2, (C) entry 5 and (D) entry 6 of Table 2.

**Table S1**. Cu-catalyzed photoinduced ATRP of different monomers, initiators and solvents using *ht*-PLP_PAN_ (0.95 mg/mL) under blue light irradiation. Vol%(monomer) was kept at 25%. [M]_0_/[I]_0_/[CuBr_2_]_0_/[TPMA]_0_ =200/1/0.02/0.06. *M*_n,GPC_ was calculated from PMMA calibrations.

| **Entry** | **Solvent** | **Initiator** | **Monomer** | **Conv. (%)** | ***M*_n,theo_** | ***M*_n,GPC_** | ***Đ*** |
| --- | --- | --- | --- | --- | --- | --- | --- |
| **1** | DMF/DMSO | EBiB | MA | 68 (25h) | 8800 | 6600 | 1.16 |
| **2** | Anisole^a^ | EBPA | MMA | 50 (33h) | 10300 | 7700 | 1.19 |

a: *ht*-PLP_PAN­_ was only able to be dispersed in the polymerization mixture but could not dissolve in anisole.

**
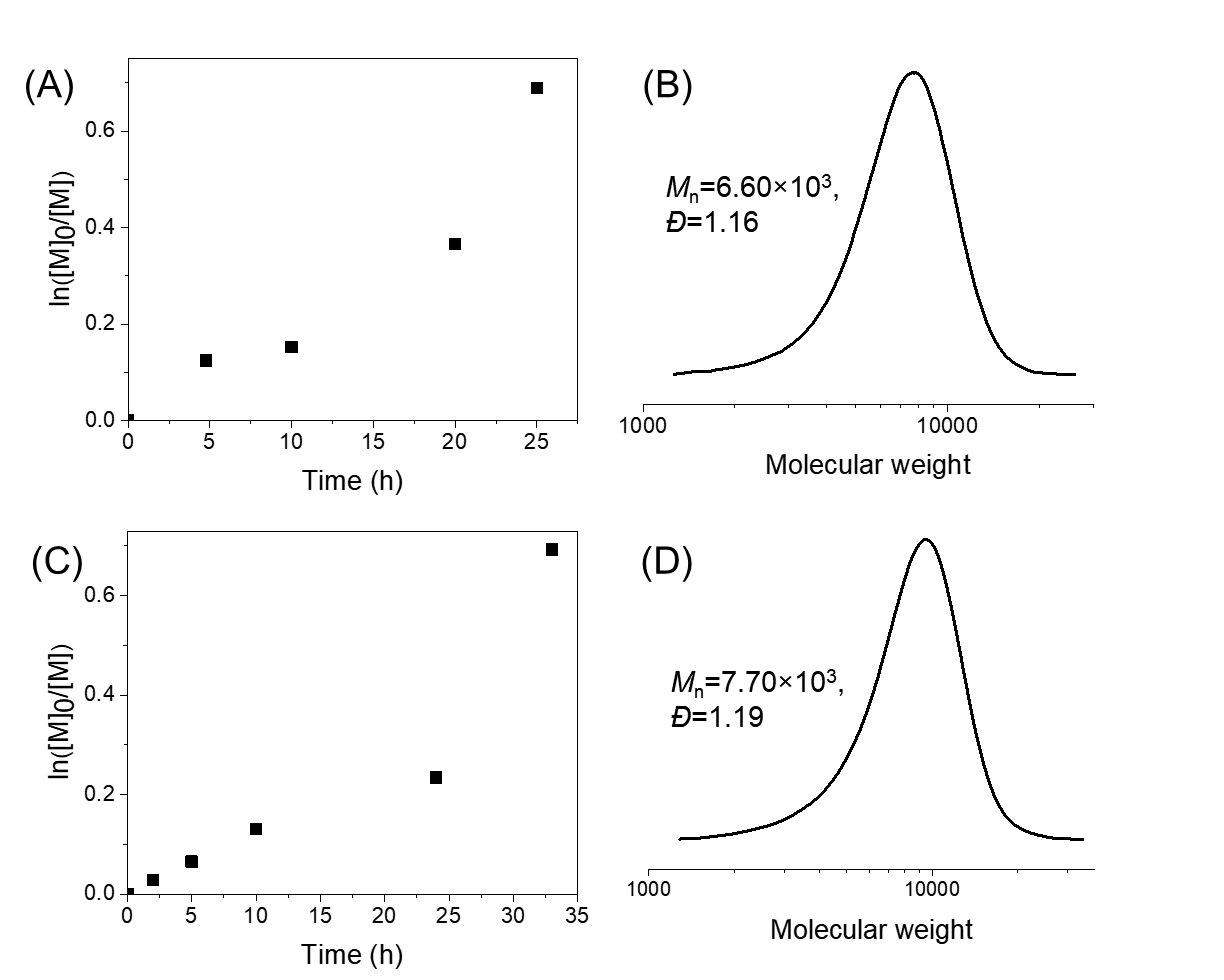
**

**Figure S6**. (A) Semilogarithmic kinetic plots corresponding to entry 1 of Table S1. (B) GPC trace of entry 1 of Table S1. (C) Semilogarithmic kinetic plots corresponding to entry 2 of Table S1. (D) GPC trace of entry 2 of Table S1.

**
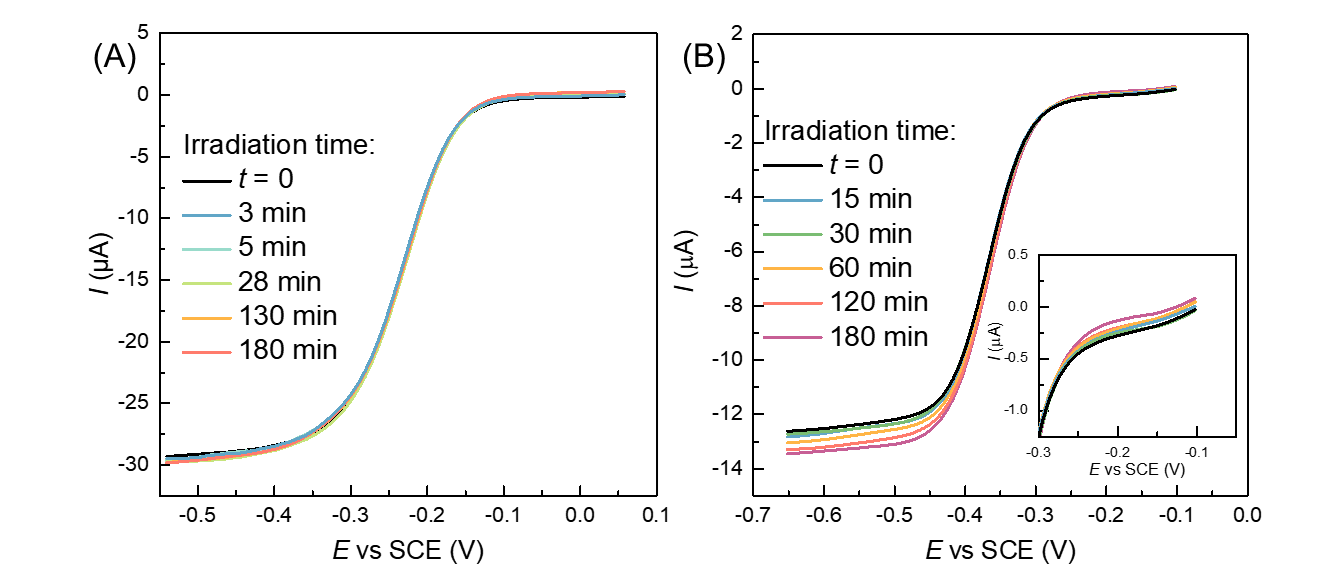
**

**Figure S7**. (A) LSV of 0.6 mM CuBr_2_/TPMA (L/Cu = 3) in DMSO-DMF 1-1 + 0.1 M Et_4_NBF_4_, in the absence of *ht*-PLP_PAN_. (B) LSV of 0.6 mM CuBr_2_/TPMA (L/Cu = 3) in water (containing 5 vol% of DMF and 60 mM NaBr) + 0.1 M Et_4_NBF_4_, in the presence of *ht*-PLP_PAN_ (1.5 mg/mL). In both instances, *v* = 0.01 Vs^-1^ and RDE rotation speed 4000 rpm were used with a blue light irradiation.

**Table S2**. Kinetic parameters for the aqueous ATRP of OEGMA using CuBr_2_/TPMA complex (molar ratio: Cu/TPMA=1/3) and HO-EBiB as the initiator. Details of scaling were discussed in Section 1.3 of Supplementary Information.

| **Coefficients** | **Referenced value** | **References and notes** |
| --- | --- | --- |
| *k*_p_ | 2.01 × 10^3^ M^-1^ s^-1^ | [*Macromol Chem Phys* **2016,** *217*, 2391-2401.](https://onlinelibrary.wiley.com/doi/full/10.1002/macp.201600302) |
| *k*_t_ | 2.74 × 10^7^ M^-1^ s^-1^ |  |
| *K*_ATRP_ | 1.8 × 10^-3^ | [*Macromolecules* **2015**, 48, 19, 6862–6875](https://pubs.acs.org/doi/10.1021/acs.macromol.5b01454) (scaled) |
| *k*_a1_ | 5.4 × 10^4^ M^-1^ s^-1^ | [*Macromolecules* **2017**, 50, 7, 2696–2705](https://pubs.acs.org/doi/full/10.1021/acs.macromol.7b00246) (scaled) |

**Table S3**. Calculated kinetic parameters for conditions corresponding to photoATRP of OEGMA catalyzed by 500 ppm of CuBr_2_/TPMA and using HO-EBiB as the initiator (entry 1, Table 1).

| **Constants** | **Calculated value** | **Definition** |
| --- | --- | --- |
| *R*_p_ (500 ppm) | 7.22 × 10^-5^ M s^-1^ | Rate of propagation, 500 ppm CuBr_2_/3×TPMA |
| *R*_p_ (control, no Cu) | 6.34 × 10^-6^ M s^-1^ | Rate of propagation, no Cu(II) complex |
| [R^●^] | 6.7 × 10^-8^ M | Concentration of propagating radical |
| *k*_a-pc_ | 6.9 × 10^-4^ cm^3^ g^-1^ s^-1^ | Apparent rate coefficient of activation by PC |
| [Cu^I^/L] | 3.6 × 10^-6^ M | Concentration of Cu(I)/L |
| Cu^I^/L % | 1.4% | Percentage of Cu(I)/L among all Cu species |
| *R*_a1_ | 5.3 × 10^-4^ M s^-1^ | Rate of activation by Cu(I) |
| *R*_a-pc_ | 9.3 × 10^-10^ M s^-1^ | Rate of activation by photocatalyst |

**Table S4**. Calculated kinetic parameters for conditions corresponding to photoATRP of OEGMA catalyzed by 100 ppm of CuBr_2_/TPMA and using HO-EBiB as the initiator (entry 2, Table 1).

| **Constants** | **Calculated value** | **Definition** |
| --- | --- | --- |
| *R*_p_ (100 ppm) | 5.46 × 10^-5^ M s^-1^ | Rate of propagation, 100 ppm CuBr_2_/3×TPMA |
| [R^●^] | 5.0 × 10^-8^ M | Concentration of propagating radical |
| [Cu^I^/L] | 5.5 × 10^-7^ M | Concentration of Cu(I)/L |
| Cu^I^/L % | 1.0% | Percentage of Cu(I)/L among all Cu species |
| *R*_a1_ | 8.1 × 10^-5^ M s^-1^ | Rate of activation by Cu(I) |


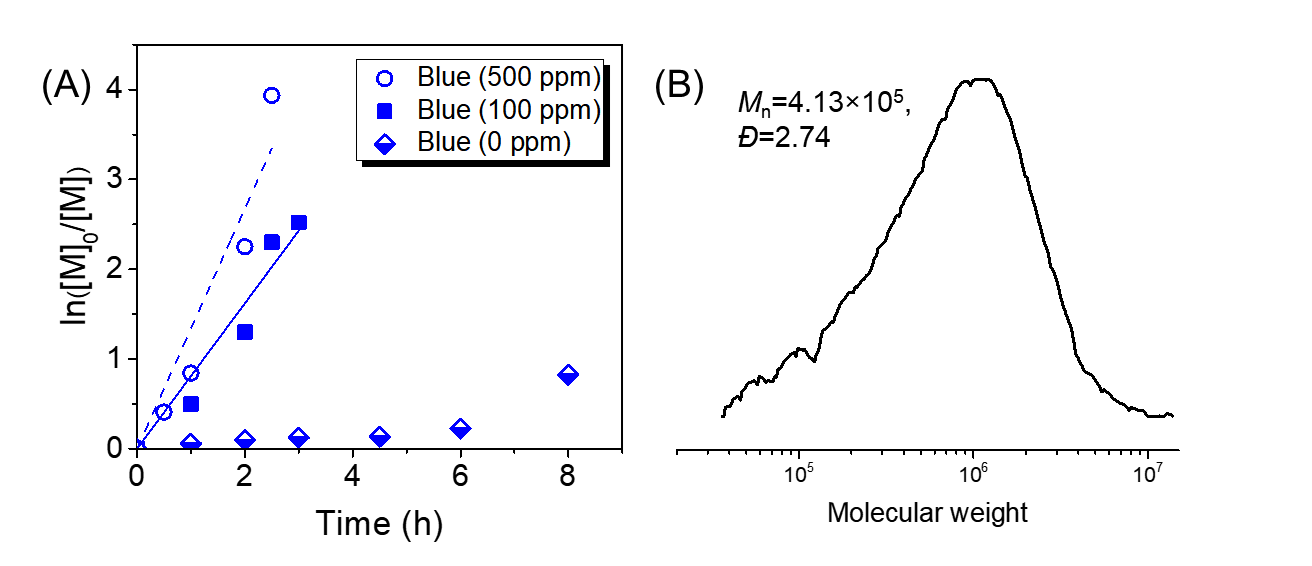


**Figure S8**. (A) Semilogarithmic kinetic plots corresponding to entries 1 (blue, 500 ppm), 2 (blue, 100 ppm) and 4 (blue, 0 ppm) of Table 1. (B) GPC trace of entry 4 of Table 1 at 8h. Molecular weight was calculated based on a PMMA calibration.

# 3. DFT Calculations and Cartesian Coordinates

**DFT calculation methods**

DFT and TDDFT calculations were performed with Gaussian 09 package Rev. A02^6^. The Optimizations and TDDFT of ***o*I_3_** to ***o*I_10_** were performed in simulated water environment using a polarizable continuum model, at the ωB97X-D/6-31g-d and cam-b3lyp/6-31g-d levels, respectively. Optimization of [Br-Cu^II^/TPMA]^+^ were performed in simulated water environment using a polarizable continuum model, at the ωB97X-D level with def2TZVP basis set on Cu and 6-31g-d basis set on all the other atoms.

**[Br-Cu^II^/TPMA]^+^**

Cu, -0.0012915052, 0.0049731859, -0.0084729205

N, -2.0808590131, -0.0100876391, -0.374857895

N, 1.0284586204, 1.8114919912, -0.3740432296

N, 1.0478208172, -1.7914877179, -0.3692494617

N, 0.0004748815, 0.0027643864, -2.089355141

C, 2.4511235791, -4.0244616005, -1.2026467387

C, 0.9639148762, -2.2056586483, -1.6438076279

C, 1.230995015, 2.8147685617, 0.4878117675

C, 1.6577604024, -3.3175286358, -2.1010224118

C, 2.530443058, -3.595016959, 0.1182540378

C, 2.046095004, 3.0991494121, -2.1077068649

C, 1.8135749882, -2.4682290047, 0.494485977

C, 1.4301092902, 1.9430662023, -1.6486442584

C, 1.848457636, 3.9983014037, 0.1096545626

C, 1.2066544759, 0.7356347799, -2.5247636795

C, 0.0330599312, -1.4086919748, -2.5232576737

C, -2.3949415003, 0.2704266297, -1.6499302214

C, -3.7035844287, 0.2239830125, -2.1103303375

C, -1.2372823549, 0.680337064, -2.5256550163

C, -3.0513063435, -0.3371117767, 0.486263267

C, -4.7141570228, -0.1118152603, -1.2145172573

C, -4.3845924526, -0.3960752015, 0.1068557867

C, 2.2607468406, 4.1416080298, -1.2112627743

H, 3.0047708717, -4.8968459671, -1.5328509146

H, 0.8848147145, 2.6414137041, 1.5013675562

H, 1.5770322927, -3.6198767306, -3.1391147186

H, 3.1399879933, -4.116356034, 0.8466008865

H, 2.349172524, 3.1788530535, -3.1456112082

H, 1.8348864993, -2.081078114, 1.5078155972

H, 1.9947918242, 4.788250148, 0.8365441004

H, 1.1330117817, 1.0139731415, -3.5821227254

H, 2.063956834, 0.0625401001, -2.4202905243

H, 0.3154634245, -1.4848198524, -3.5791667235

H, -0.9788724713, -1.8146841158, -2.4227094805

H, -3.9233467497, 0.4456131658, -3.1487531957

H, -1.4414979015, 0.4759881211, -3.5826437776

H, -1.0832045656, 1.759562224, -2.4223950821

H, -2.7288585603, -0.5492653392, 1.5002697727

H, -5.7456707504, -0.1558316329, -1.5471961644

H, -5.1421912853, -0.6647864268, 0.8332421523

H, 2.7397676373, 5.0564912074, -1.5431769502

Br, -0.0043719077, 0.0083771065, 2.3780505105

***o*I_3_**

N, 3.8130449526, -0.4107677022, 1.4629231796

C, 3.3718174645, -0.7162062644, 0.0979478672

N, 2.0517178907, -1.3335815781, 0.2005753784

C, 1.0150528587, -0.6779207732, -0.1613452125

N, -0.2105105295, -1.3745542426, -0.0601338985

C, 3.440213732, 0.4634372069, -0.8846045946

C, 0.9725423313, 0.7296378976, -0.7273891034

C, 2.314015311, 1.4466986013, -0.5995841407

C, -0.1681150629, 1.4843569736, -0.0435183967

C, -1.4984847447, 0.7648818421, -0.240784032

C, -2.5917778959, 1.2548679762, 0.7246098116

C, -2.8569403703, 2.7560746859, 0.622555782

H, 2.4192093299, 1.8511172622, 0.4165126839

H, 2.344937991, 2.3046933464, -1.2791484635

H, 0.7252490107, 0.6275002668, -1.7955383083

H, 0.0530090201, 1.5650513734, 1.0308961025

H, -3.5175840783, 0.7109337802, 0.5098468381

H, -2.3023625075, 0.9988149611, 1.7525487272

H, -3.6964481515, 3.0444969256, 1.2632274429

H, 3.343282484, 0.0813650604, -1.9096706529

H, -1.8566867643, 0.9453894015, -1.2648727735

H, -0.2176296281, 2.5062359027, -0.4349953604

H, -3.1054527886, 3.0394079663, -0.4070739767

C, -1.3263665147, -0.7401651194, -0.1394767923

O, -3.2544115905, -1.3984861503, -1.2752605136

C, -2.6119304494, -1.5741628893, -0.2119723517

O, -2.8777362809, -2.3120220561, 0.7589173676

H, -1.9869610405, 3.3447736419, 0.9316872527

H, 4.0469180724, -1.5014204042, -0.2655659715

H, 4.4227715292, 0.9427154309, -0.8098258884

H, 3.2324903256, 0.3348404913, 1.8426210434

H, 4.7598630941, -0.0390098144, 1.425110954

***o*I_4_**

C, -4.2928849557, 1.1014558213, -0.3587697332

C, -4.3159261848, -0.3001505726, -0.9713024209

N, -3.0237169526, -0.9811394805, -0.9594103326

C, -2.1468345762, -0.6302671135, -0.0985342172

N, -0.9064535339, -1.2922155145, -0.1723399893

C, 0.1452630682, -0.6732936049, 0.2347397752

N, 1.3462988536, -1.3906174602, 0.1969709316

C, -2.2825634939, 0.4303569315, 0.9788862139

C, 0.1975989188, 0.7402129493, 0.7770074318

C, -3.6822048975, 1.0480565554, 1.0353234368

C, -1.1575217505, 1.4411210348, 0.7142658314

C, 1.3034312443, 1.477553651, 0.0179383018

C, 2.6411156204, 0.7526383229, 0.1591682353

C, 3.6460378847, 1.1384702171, -0.941482582

C, 3.9313969875, 2.6382414052, -0.9914629093

H, -4.3250944859, 0.4340735091, 1.6728333519

H, -3.6293645174, 2.0456746588, 1.4854018306

H, -5.3099506332, 1.5086289804, -0.3280407942

H, -1.299651176, 1.8976443779, -0.2745080272

H, -1.1921188658, 2.251343466, 1.4503387739

H, 0.5013759552, 0.6501330189, 1.8308284792

H, 1.0226387479, 1.5392984383, -1.0430846318

H, 4.5838905602, 0.5982869598, -0.7741287081

H, 3.2556472814, 0.8018357335, -1.9109096888

H, 4.6953792289, 2.8601326895, -1.7432284218

H, -2.0659409996, -0.0596652487, 1.9378888346

H, 3.0936524407, 1.0122028733, 1.127008541

H, 1.3813760515, 2.5057010454, 0.3868075682

H, 4.2957524246, 2.999994309, -0.02281012

H, -3.7040765834, 1.7568029919, -1.0131287849

C, 2.4665774291, -0.7527726221, 0.2128387894

H, -4.5825894506, -0.2056428084, -2.0361724121

O, 4.3452254696, -1.3598725833, 1.4457070075

C, 3.7543973869, -1.5676820073, 0.3604081553

O, 4.0706466183, -2.3128649538, -0.5888404906

H, 3.0356966969, 3.2129296634, -1.2496877469

N, -5.2699529106, -1.1525842627, -0.2518364482

H, -6.2136505966, -0.8323446157, -0.4615459272

H, -5.2030973046, -2.0921957557, -0.6379461033

***o*I_5_**

C, 5.5360684262, 1.0163738244, 0.391768602

C, 5.4582340673, -0.3065140918, 1.1559156361

N, 4.1331945214, -0.921142429, 1.1564292031

C, 3.3141581026, -0.6294042717, 0.2198386525

N, 2.0359589453, -1.2120607494, 0.3174263731

C, 1.0339794349, -0.6088825314, -0.2205080879

N, -0.1971995788, -1.2594182643, -0.1223450407

C, 3.5543073875, 0.2901776187, -0.9629795902

C, 1.0896132189, 0.713277979, -0.9648981165

C, 4.9832839689, 0.8367141687, -1.0156610509

C, 2.4647887529, 1.3679825432, -0.8758342987

C, -0.0457573496, 1.5913821484, -0.4398160528

C, -1.3840336233, 0.8863179529, -0.6239211064

C, -2.4947054933, 1.4615318803, 0.2575186579

H, 5.6220446415, 0.1294619206, -1.5523875231

H, 4.9993258657, 1.7807951139, -1.5715457924

H, 6.5742300605, 1.3667135174, 0.3646791212

H, 2.5617377784, 1.9148319499, 0.0715963118

H, 2.5813604649, 2.098504252, -1.6835385908

H, 0.8832800227, 0.4766145476, -2.0201464152

H, 0.1239505553, 1.7951432657, 0.6260414944

H, -2.2049973885, 1.3526861619, 1.3121247886

H, 3.3604593182, -0.2976954003, -1.8705362385

H, -1.6937374058, 0.9751719426, -1.6763758299

H, -0.0526586365, 2.5573552429, -0.9555732111

H, 4.957419179, 1.7698002817, 0.9412304365

C, -1.2853918208, -0.5958513957, -0.3349197678

H, 5.6821668527, -0.1058132767, 2.2159269762

N, 6.3973527488, -1.2786818637, 0.5837944523

H, 7.3459484826, -0.9821314686, 0.8057219549

H, 6.2635857405, -2.1651961129, 1.0662666649

H, -2.5970180932, 2.5339560684, 0.0628878086

C, -3.8155518473, 0.7389781555, 0.0086883179

H, -4.2549887481, 1.104217053, -0.9315027437

N, -2.4633062228, -1.342391827, -0.3240996589

C, -3.6028191871, -0.7431881805, -0.2223626921

C, -4.866475985, -1.5855048784, -0.4116172743

O, -5.0985312896, -2.4594240336, 0.4468633909

O, -5.5226504974, -1.2645554226, -1.4290770335

C, -4.8496170753, 0.9740482564, 1.1242809697

H, -5.772328164, 0.4436762628, 0.8667342233

H, -4.4761144608, 0.5293067755, 2.0558622924

C, -5.1645783064, 2.4522363082, 1.3465261738

H, -4.2894653144, 3.0054692577, 1.7026904103

H, -5.5058860322, 2.9232793756, 0.417348293

H, -5.9557520164, 2.5682173728, 2.0939299101

***o*I_6_**

C, 6.7238708736, 0.9087442972, 0.5074513216

C, 6.5344654431, -0.3180010861, 1.4008352774

N, 5.17862675, -0.8603168142, 1.3905524281

C, 4.4313636426, -0.6387746892, 0.3779060823

N, 3.1204092563, -1.1455190547, 0.4611915357

C, 2.1839108918, -0.5653822266, -0.2048435011

N, 0.9168306628, -1.1410767746, -0.1030506109

C, 4.7889153748, 0.1244439886, -0.8844391099

C, 2.351713471, 0.6527272239, -1.0945872648

C, 6.244275983, 0.6013765765, -0.9048689832

C, 3.7522055218, 1.2507351632, -0.9985966445

C, 1.2355814772, 1.6375469781, -0.7483756285

C, -0.1239078151, 0.9764553756, -0.9382019091

C, -1.2581406027, 1.7117371008, -0.2243893016

H, 6.8779499401, -0.1861045765, -1.3227699951

H, 6.3381514114, 1.4780880239, -1.5553562555

H, 7.7786083432, 1.2066996534, 0.5083816808

H, 3.8224747503, 1.9077980561, -0.1212673531

H, 3.9532885547, 1.867922841, -1.8807094878

H, 2.1927227657, 0.2994563263, -2.1251919588

H, 1.352009998, 1.9590374278, 0.2954225078

H, -1.051252682, 1.7249513178, 0.8541856396

H, 4.6228308517, -0.559662694, -1.7279139629

H, -0.3543605994, 0.9394088724, -2.0139824616

H, 1.3090253809, 2.5345435258, -1.3721856305

H, 6.1540220724, 1.7425499233, 0.9369487927

C, -0.1252385616, -0.4621282915, -0.4568517349

H, 6.7172164574, -0.0160638454, 2.4445976031

N, 7.4435086284, -1.3932110487, 0.9861834247

H, 8.3956277772, -1.1231929064, 1.2261327848

H, 7.2367707209, -2.2138517088, 1.5521466702

H, -1.3040617146, 2.7541230883, -0.5562192983

C, -2.5873043477, 1.0156924478, -0.4851435661

H, -2.9431990091, 1.28255734, -1.4918279483

N, -1.3326676173, -1.148631553, -0.4126199132

C, -2.4459661641, -0.4895160267, -0.4702868729

C, -3.6710709874, 1.3903220742, 0.5288821477

H, -3.3304092541, 1.1051563316, 1.5341615901

N, -3.6015681163, -1.2575113633, -0.559384128

C, -4.7507244365, -0.718481965, -0.3130532144

C, -4.984957137, 0.6835433769, 0.2085152766

H, -5.4818403704, 1.2044365305, -0.6236262267

H, -3.8060485322, 2.4765921062, 0.533188374

C, -6.0021381479, -1.5380913838, -0.6262235836

O, -6.2225177818, -2.5277356661, 0.0994599131

O, -6.6614179226, -1.0789677904, -1.5861944409

C, -5.9644054995, 0.6680725585, 1.3961103378

H, -6.8856499964, 0.1618772977, 1.0893311447

H, -5.5301173328, 0.0703754955, 2.2079220304

C, -6.3039712633, 2.0670771694, 1.9067090457

H, -7.0584279247, 2.0144295083, 2.6978344158

H, -5.4253996577, 2.573194851, 2.3199758082

H, -6.7037635261, 2.6915456176, 1.0993071534

***o*I_7_**

C, 7.9658299702, 0.4489754536, 0.7181524955

C, 7.6686989907, -0.9051406232, 1.363439439

N, 6.2729388216, -1.3212150511, 1.2567686448

C, 5.5614821022, -0.8577290515, 0.3015610547

N, 4.2126147312, -1.2606866133, 0.2931152838

C, 3.3359507401, -0.5033350592, -0.2687589711

N, 2.0268056523, -0.985365159, -0.2677525763

C, 6.000825263, 0.0877773487, -0.8019824482

C, 3.6157332937, 0.8282888626, -0.9409789493

C, 7.4877653076, 0.4495002961, -0.7275734964

C, 5.0526800951, 1.2923126743, -0.7248544964

C, 2.5643686493, 1.8260448844, -0.4576169278

C, 1.1682585343, 1.3087158874, -0.7800058106

C, 0.064041336, 2.0158433197, 0.0044361669

H, 8.0690952463, -0.2907518627, -1.284373355

H, 7.6564752169, 1.4231798274, -1.2008851608

H, 9.0399871847, 0.6586931192, 0.7768343057

H, 5.1496346519, 1.7773303704, 0.2558624459

H, 5.3193321661, 2.0378827159, -1.4814770656

H, 3.4620200761, 0.6615608174, -2.018497359

H, 2.67070296, 1.9641186016, 0.6268172661

H, 0.2269811141, 1.8527128344, 1.0782453192

H, 5.8029292205, -0.4225816021, -1.754644819

H, 0.9771635301, 1.4502020429, -1.8549545957

H, 2.7212712635, 2.8040528457, -0.9245374362

H, 7.4537596942, 1.2287685916, 1.2961812997

C, 1.0460134375, -0.1809366105, -0.5221820915

H, 7.8647062596, -0.8229027065, 2.4445035314

N, 8.4911665007, -1.9531617297, 0.7469414261

H, 9.4602093099, -1.8105185396, 1.0256557393

H, 8.2148706478, -2.8460395907, 1.1505820212

H, 0.1069848154, 3.0961654194, -0.1679995423

C, -1.3005025426, 1.4730389405, -0.3982171857

H, -1.5784688517, 1.8927473257, -1.376997487

N, -0.2082114027, -0.7717548238, -0.5886178673

C, -1.2737998827, -0.032729289, -0.5754335934

C, -2.4004543857, 1.8106683121, 0.6093231161

H, -2.1316170819, 1.3797431234, 1.5829147317

N, -2.468283192, -0.7011326443, -0.7798757678

C, -3.5872782429, -0.1005639846, -0.5143270232

C, -3.7394947191, 1.2476025792, 0.1495899995

H, -4.1831157177, 1.9192730333, -0.5994881475

H, -2.4694799639, 2.8945729152, 0.7459628143

C, -4.7340654293, 1.0561492466, 1.2986653782

H, -4.278765702, 0.3834076891, 2.0382360093

H, -4.9065319281, 2.0123002966, 1.8041336552

N, -4.7439193076, -0.7487057025, -0.911003178

C, -6.0644258676, 0.4685400165, 0.8225306804

H, -6.7125322765, 1.2747211167, 0.4531024299

C, -5.8799194748, -0.4392136669, -0.3749648973

C, -7.1352315054, -0.9479267598, -1.0872737422

O, -7.3223407671, -2.1804551605, -1.1203145644

O, -7.8192241756, -0.0155787328, -1.5652856112

C, -6.7864516593, -0.2180968086, 2.0027535876

H, -6.7149487226, 0.4743150044, 2.8509592411

H, -6.2162389796, -1.1114077771, 2.2893622636

C, -8.2532283314, -0.5858057295, 1.7842170973

H, -8.3586795544, -1.3985732393, 1.0619988643

H, -8.6998770331, -0.9131417792, 2.7288301063

H, -8.8250730858, 0.2735497849, 1.4181357521

***o*I_8_**

C, 9.094106937, 0.3605548152, 0.8178877804

C, 8.726572534, -0.8742952653, 1.6417758736

N, 7.3197707282, -1.2550265601, 1.548145603

C, 6.653273867, -0.9090324798, 0.5141504447

N, 5.2903868504, -1.2626934782, 0.5190598331

C, 4.4595872789, -0.5627683492, -0.1718240355

N, 3.1325654727, -0.9932284926, -0.1422854558

C, 7.1581243643, -0.1462538129, -0.6974180704

C, 4.8088984884, 0.6482337303, -1.0172250487

C, 8.6556346791, 0.1691331029, -0.6277988156

C, 6.2559038057, 1.0893141565, -0.8211146425

C, 3.7824506531, 1.7399066345, -0.718621872

C, 2.378465658, 1.227609995, -1.0136233381

C, 1.2759136149, 2.0820641176, -0.3903977078

H, 9.2213663342, -0.6638671615, -1.0546195067

H, 8.8757020842, 1.0577446503, -1.2298515958

H, 10.1736217809, 0.5390257229, 0.8808359228

H, 6.344019312, 1.7046943799, 0.0844008481

H, 6.5718023229, 1.7106775865, -1.6658428551

H, 4.6803822598, 0.3330285203, -2.0644588433

H, 3.8605494102, 2.0293158216, 0.3380336837

H, 1.3919368033, 2.079322105, 0.7017296375

H, 6.965318515, -0.7797382789, -1.5742020705

H, 2.2298411408, 1.2110510882, -2.1044540386

H, 3.9905388338, 2.6344286457, -1.3150096387

H, 8.597605803, 1.2333720877, 1.2607139342

C, 2.1909161434, -0.2013614306, -0.5413283885

H, 8.8930308688, -0.6412962692, 2.7056938447

N, 9.5265996399, -2.0280009557, 1.2133544425

H, 10.4913101719, -1.8792047459, 1.503575056

H, 9.2035955954, -2.8429298871, 1.7310720792

H, 1.3666404962, 3.1213767028, -0.7224506111

C, -0.0915042708, 1.5279247633, -0.7659739266

H, -0.3032232113, 1.7840673903, -1.8156276092

N, 0.9160600043, -0.7521810359, -0.5513623445

C, -0.1209084426, 0.0146654369, -0.6862702246

C, -1.2244604216, 2.0712008823, 0.1034466504

H, -1.0365409916, 1.7975989211, 1.1503292084

N, -1.3341696612, -0.6442090382, -0.7926848596

C, -2.4444928711, 0.0205052314, -0.684385918

C, -2.5585775371, 1.4938391522, -0.3486618382

H, -2.8698776883, 1.9966028125, -1.2771882161

H, -1.2497694854, 3.1645255597, 0.0547119501

C, -3.6687309761, 1.6565615593, 0.6898727611

H, -3.3735657269, 1.1375577542, 1.6115987071

H, -3.7992442332, 2.7135979679, 0.9427465837

C, -4.9744877342, 1.0734304128, 0.1666156468

N, -3.5959164032, -0.7031251758, -0.93327433

C, -4.7491029102, -0.2105262029, -0.5971770303

N, -5.8569486967, -0.9315862617, -1.0146837576

H, -5.4266381874, 1.7803434794, -0.5455033688

C, -5.9901644411, 0.7817115107, 1.2747321052

H, -6.1878259346, 1.6997725233, 1.8369043015

H, -5.5505948223, 0.0618243096, 1.9791679627

C, -7.2825931615, 0.2137326607, 0.6953755702

H, -7.8773281094, 1.0332793539, 0.264172643

C, -7.0121699514, -0.703214295, -0.4776306009

C, -8.2332772503, -1.3676709872, -1.1111328027

O, -8.3285183503, -2.6038207729, -0.9820774716

O, -8.998339293, -0.5522000143, -1.6728889746

C, -8.157597024, -0.488444592, 1.7495884678

H, -9.0698030268, -0.8536102517, 1.2662852091

H, -7.6232939458, -1.3704343279, 2.1255259203

C, -8.5347859876, 0.4252291793, 2.9141129746

H, -9.2164873127, -0.0881792466, 3.5992783296

H, -7.6561330293, 0.7333325078, 3.4901882503

H, -9.0361993626, 1.3310291375, 2.5539045826

***o*I_9_**

C, 10.2771889981, -0.2438056293, -0.8014837743

C, 9.8724458577, 0.9037053226, -1.7279551051

N, 8.4551848158, 1.2498589084, -1.6636499355

C, 7.7984578618, 0.9731024628, -0.6030583666

N, 6.4259478608, 1.2861037656, -0.6347099773

C, 5.6155776162, 0.6195549622, 0.1110668774

N, 4.276170132, 1.0066682186, 0.0486369517

C, 8.3237623843, 0.3287496009, 0.6669404383

C, 5.9992205192, -0.5094439138, 1.0498625248

C, 9.830516493, 0.0561732974, 0.6231586956

C, 7.4597641899, -0.9201865946, 0.8905970074

C, 5.0069165798, -1.6517755507, 0.8375977084

C, 3.5878244115, -1.1601330839, 1.0927711538

C, 2.5115795002, -2.0938677536, 0.5416089909

H, 10.3690337392, 0.939940347, 0.9771915562

H, 10.0774594536, -0.7706176129, 1.2984038389

H, 11.3618925547, -0.3928835795, -0.8501247375

H, 7.5691977316, -1.6039710479, 0.0380452469

H, 7.7923900004, -1.4608686343, 1.7830420062

H, 5.8576171369, -0.1166495332, 2.0687680148

H, 5.0955449381, -2.0202707497, -0.1932913798

H, 2.6267571217, -2.1740641692, -0.5476743639

H, 8.1088754252, 1.0252367235, 1.4890492646

H, 3.4383369399, -1.0613175504, 2.1792341309

H, 5.2405466811, -2.4902645576, 1.5020260518

H, 9.8092805522, -1.1666088488, -1.167458563

C, 3.3581072888, 0.2206132361, 0.5088300832

H, 10.0451957817, 0.5843950824, -2.7682201902

N, 10.6377167765, 2.113542486, -1.40370619

H, 11.6060946323, 1.9695314651, -1.6839307321

H, 10.289176041, 2.8707739076, -1.9881498084

H, 2.6331979031, -3.1004597801, 0.9547213065

C, 1.1288937377, -1.5521662944, 0.8766083534

H, 0.9290817424, -1.725273519, 1.945546069

N, 2.0667253669, 0.7319567523, 0.4743013283

C, 1.0532544044, -0.0524982208, 0.6708809773

C, 0.0095845973, -2.2010058785, 0.0642914895

H, 0.1813808978, -2.0097868666, -1.0033888146

N, -0.179965389, 0.5766624267, 0.718400049

C, -1.2701271001, -0.1274954466, 0.6725180784

C, -1.3380018597, -1.628838326, 0.4806202907

H, -1.6175240833, -2.0505376158, 1.4585294394

H, 0.0188566176, -3.2866566126, 0.2043598949

C, -2.4573467781, -1.9297097292, -0.5148364698

H, -2.2020082615, -1.4890826176, -1.4877083543

H, -2.5521048588, -3.010014661, -0.6636897862

C, -3.77293944, -1.3503137339, -0.0147333562

N, -2.4415098449, 0.5872066092, 0.8445000649

C, -3.5852099621, 0.032077171, 0.5754842155

N, -4.6973561221, 0.7888201533, 0.8939346867

H, -4.1527075626, -1.9822824463, 0.8030984915

C, -4.8476852481, -1.2603932698, -1.0976436043

H, -5.0295045946, -2.2477564498, -1.5340044427

H, -4.4900799395, -0.6091051624, -1.906274267

C, -6.1355769552, -0.6983860661, -0.5123466854

H, -6.6194465458, -1.4830705688, 0.0884793311

C, -5.8640502274, 0.4441956386, 0.4395184344

C, -7.1218372412, -0.2103531887, -1.5777148848

H, -6.643857005, 0.5870001633, -2.1598040396

N, -6.9316020707, 1.1960188381, 0.9114750556

H, -7.3392357019, -1.0248256405, -2.2758317719

C, -8.3984086121, 0.3165861459, -0.9239924323

H, -8.908766413, 1.0119888979, -1.6011684912

C, -8.0805784714, 1.1177214914, 0.3193045238

C, -9.2313882941, 1.9377265746, 0.8989749335

O, -9.5169499955, 2.9408051108, 0.2091429887

O, -9.7474814289, 1.5116738438, 1.9520813631

C, -9.4109153221, -0.7967561926, -0.5602288845

H, -8.8988103345, -1.5810635729, 0.0115208279

H, -10.1701388693, -0.3798787867, 0.1090812706

C, -10.093274091, -1.406285971, -1.7831755842

H, -10.8138245581, -2.1714578912, -1.4776992422

H, -10.6339622298, -0.6395666948, -2.3492370145

H, -9.3757818709, -1.8809445903, -2.4605417557

***o*I_10_**

C, 10.8634950483, 0.0696141325, 0.4905843469

C, 10.4948102851, -1.0585461967, 1.4653101022

N, 9.0669675427, -1.3708241805, 1.4780121493

C, 8.3609511203, -1.0949592698, 0.448641018

N, 6.9842123003, -1.3697239388, 0.5614808695

C, 6.1506757061, -0.6994083858, -0.1551563912

N, 4.8072159061, -1.0467158169, -0.0071927148

C, 8.8311266304, -0.4923899109, -0.8643044672

C, 6.5113752001, 0.3949241398, -1.1429416014

C, 10.3431140763, -0.250424558, -0.9043335198

C, 7.9872604971, 0.7725810177, -1.0713811

C, 5.5600489545, 1.5674090046, -0.9111428788

C, 4.1192120578, 1.1047561937, -1.082218815

C, 3.0932449484, 2.0850355335, -0.5176997793

H, 10.8495549276, -1.1448947732, -1.2858632973

H, 10.5728341638, 0.5566917315, -1.6082606451

H, 11.9503807616, 0.2078426906, 0.4843672733

H, 8.1587037693, 1.4723838546, -0.2424124698

H, 8.2844413894, 1.2842054427, -1.9929529202

H, 6.3069321978, -0.0201078718, -2.1421272411

H, 5.7094767917, 1.9594685109, 0.1039486577

H, 3.2590720845, 2.2010738029, 0.561783557

H, 8.5577868337, -1.2013631403, -1.6576646128

H, 3.9170346552, 0.9723897304, -2.1565997492

H, 5.7796895082, 2.3825461867, -1.6086325674

H, 10.4213103496, 1.0072324753, 0.8523655459

C, 3.884616578, -0.2495145364, -0.4402363564

H, 10.7401943673, -0.723030926, 2.4806191839

N, 11.2216177672, -2.3164110402, 1.2735963934

H, 11.0514049172, -2.6655627399, 0.332439308

H, 12.2206956065, -2.1302198651, 1.329921662

H, 3.2200261139, 3.0727926428, -0.9727128935

C, 1.6852083289, 1.5665300521, -0.7721890639

H, 1.4492303966, 1.6934808225, -1.840296191

N, 2.5849298117, -0.722101532, -0.3139606131

C, 1.580737091, 0.0804369282, -0.491809212

C, 0.6151104043, 2.2852237705, 0.0464261634

H, 0.8200609074, 2.1415559644, 1.1157103377

N, 0.3332791715, -0.5163546373, -0.4419073249

C, -0.7391016477, 0.2177298181, -0.3921271493

C, -0.7600023266, 1.7300053379, -0.2948567097

H, -1.0538441828, 2.0968977393, -1.290770635

H, 0.6472941494, 3.3620827823, -0.1479980083

C, -1.8415127875, 2.129852946, 0.7067184213

H, -1.5729606119, 1.7462828182, 1.7000265408

H, -1.9011992989, 3.2200395086, 0.7856244615

C, -3.1858404537, 1.5591663957, 0.2788268078

N, -1.9303525101, -0.4750522937, -0.4655789549

C, -3.0542431973, 0.1301338898, -0.2082764711

N, -4.1903572927, -0.6133661097, -0.43903104

H, -3.5635086599, 2.1406723534, -0.5764889017

C, -4.2362271474, 1.5882143417, 1.3880073257

H, -4.3774756084, 2.6116052179, 1.7498868856

H, -3.8785407266, 0.9905220558, 2.2368552528

C, -5.5547669204, 1.0243036972, 0.877254032

H, -6.0321788232, 1.7762321477, 0.2312781836

C, -5.3418265218, -0.1956810198, 0.0052479334

C, -6.5212504742, 0.6512213412, 2.0061554859

H, -6.0312211097, -0.0803100802, 2.659206751

N, -6.4372565731, -0.919566219, -0.404269319

H, -6.7351105713, 1.5350886127, 2.6162178627

C, -7.813601618, 0.0611412488, 1.452013594

H, -8.2854448265, -0.5832917629, 2.2021158675

C, -7.5711116692, -0.7661826162, 0.2133116815

C, -8.8421036773, 1.1174876451, 1.001835784

N, -8.6681835569, -1.4194268143, -0.2937819385

H, -9.1266815639, 1.7307703714, 1.8622632553

H, -8.3646061508, 1.7901889171, 0.2777609801

C, -10.0993193483, 0.4902621145, 0.375034328

H, -10.8668951717, 0.3541730273, 1.1460005247

C, -9.8441592507, -0.9080583541, -0.1473528155

C, -11.051980905, -1.813504259, -0.4212602828

O, -11.7926811429, -1.9328953761, 0.5802588237

O, -11.1477354415, -2.3212104817, -1.5557524583

C, -10.675701778, 1.4274174633, -0.7086884794

H, -10.7379961493, 2.4261177876, -0.2587950478

H, -9.9468965919, 1.5026690194, -1.5264364838

C, -12.0434667763, 1.0382125506, -1.2659837293

H, -12.416080294, 1.8235237872, -1.9314642532

H, -11.9923340382, 0.1069213476, -1.8361384573

H, -12.7721214708, 0.901287645, -0.4598129313

# REFERENCES

1. Kopec, M.; Yuan, R.; Gottlieb, E.; Abreu, C. M. R.; Song, Y.; Wang, Z.; Coelho, J. F. J.; Matyjaszewski, K.; Kowalewski, T., Polyacrylonitrile-b-poly(butyl acrylate) Block Copolymers as Precursors to Mesoporous Nitrogen-Doped Carbons: Synthesis and Nanostructure. *Macromolecules* **2017,** *50*, 2759-2767.

2. Lamson, M.; Kopec, M.; Ding, H.; Zhong, M.; Matyjaszewski, K., Synthesis of Well-Defined Polyacrylonitrile by ICAR ATRP with Low Concentrations of Catalyst. *J. Polym. Sci. A Polym. Chem.* **2016,** *54*, 1961-1968.

3. Smolne, S.; Weber, S.; Buback, M., Propagation and Termination Kinetics of Poly(Ethylene Glycol) Methyl Ether Methacrylate in Aqueous Solution. *Macromol. Chem. Phys.* **2016,** *217*, 2391-2401.

4. Fantin, M.; Isse, A. A.; Gennaro, A.; Matyjaszewski, K., Understanding the Fundamentals of Aqueous ATRP and Defining Conditions for Better Control. *Macromolecules* **2015,** *48*, 6862-6875.

5. Fantin, M.; Isse, A. A.; Matyjaszewski, K.; Gennaro, A., ATRP in Water: Kinetic Analysis of Active and Super-Active Catalysts for Enhanced Polymerization Control. *Macromolecules* **2017,** *50*, 2696-2705.

6. Frisch, M. J.; Trucks, G. W.; Schlegel, H. B.; Scuseria, G. E.; Robb, M. A.; Cheeseman, J. R.; Scalmani, G.; Barone, V.; Mennucci, B.; Petersson, G. A.; Nakatsuji, H.; Caricato, M.; Li, X.; Hratchian, H. P.; Izmaylov, A. F.; Bloino, J.; Zheng, G.; Sonnenberg, J. L.; Hada, M.; Ehara, M.; Toyota, K.; Fukuda, R.; Hasegawa, J.; Ishida, M.; Nakajima, T.; Honda, Y.; Kitao, O.; Nakai, H.; Vreven, T.; Montgomery, J. A., Jr.; Peralta, J. E.; Ogliaro, F.; Bearpark, M.; Heyd, J. J.; Brothers, E.; Kudin, K. N.; Staroverov, V. N.; Kobayashi, R.; Normand, J.; Raghavachari, K.; Rendell, A.; Burant, J. C.; Iyengar, S. S.; Tomasi, J.; Cossi, M.; Rega, N.; Millam, J. M.; Klene, M.; Knox, J. E.; Cross, J. B.; Bakken, V.; Adamo, C.; Jaramillo, J.; Gomperts, R.; Stratmann, R. E.; Yazyev, O.; Austin, A. J.; Cammi, R.; Pomelli, C.; Ochterski, J. W.; Martin, R. L.; Morokuma, K.; Zakrzewski, V. G.; Voth, G. A.; Salvador, P.; Dannenberg, J. J.; Dapprich, S.; Daniels, A. D.; Farkas, Ö.; Foresman, J. B.; Ortiz, J. V.; Cioslowski, J.; Fox, D. J., Gaussian 09 Rev. A.02, Wallingford, CT, 2016.
